# Bioprospecting of Labdane‐Type Diterpenes From *Austroeupatorium laetevirens*: Molecular Networking, Structural Elucidation, and Biological Activity Evaluation

**DOI:** 10.1002/cbdv.202502381

**Published:** 2025-10-10

**Authors:** Francielli A. P. Valeze, Maria Eduarda V. de Souza, Beatriz P. Moreno, Bianca D. B. Sahm, Andressa L. Ieque, Cleverton S. Fernandes, Regiane B. L. Scodro, Leticia V. Costa‐Lotufo, Marta R. B. do Carmo, Andrea N. L. Batista, João M. Batista Junior, Ernani A. Basso, Maria H. Sarragiotto, Debora C. Baldoqui

**Affiliations:** ^1^ Departamento de Química Universidade Estadual de Maringá Maringá Paraná Brazil; ^2^ Instituto de Ciências Biomédicas Universidade de São Paulo São Paulo São Paulo Brazil; ^3^ Laboratório de Bacteriologia Médica Universidade Estadual de Maringá Maringá Paraná Brazil; ^4^ Departamento de Biologia Geral Universidade Estadual de Ponta Grossa Ponta Grossa Paraná Brazil; ^5^ Instituto de Química Universidade Federal Fluminense Niterói Rio de Janeiro Brazil; ^6^ Instituto de Ciência e Tecnologia Universidade Federal de São Paulo São José dos Campos São Paulo Brazil

**Keywords:** Asteraceae. *Austroeupatorium laetevirens*. dereplication study. labdane‐type diterpenes. NMR spectroscopy. one new labdane‐type

## Abstract

Labdane‐type diterpenes are the main specialized metabolites in the *Austroeupatorium* genus, known for diverse biological activities. This study employed molecular networking to facilitate the identification of labdanes in *Austroeupatorium laetevirens*, alongside isolation, structural elucidation, and evaluation of anti‐*Mycobacterium tuberculosis* and antiproliferative activities. Ultra‐high performance liquid chromatography coupled with high‐resolution tandem mass spectrometry was used for dereplication, with data processed through the Global Natural Products Social Molecular Networking platform and visualized in Cytoscape. Structural elucidation was based on nuclear magnetic resonance spectroscopy, vibrational circular dichroism spectroscopy, and density functional theory calculations. Dereplication analysis led to the putative annotation of nine labdanes, two of which were isolated, including one new compound. Seven known compounds were also isolated, with eupatorin and eupafolin showing moderate antimycobacterial activity. Additionally, 25 compounds were putatively identified through dereplication. These findings reinforce the relevance of labdane‐type diterpenes within the *Austroeupatorium* genus.

## Graphical abstract

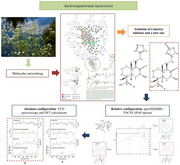

## 1 Introduction

*Austroeupatorium* R.M.King & H. Rob. is a segregated genus of *Eupatorium* that comprises 15 species, of which only two have been chemically investigated: *A. inulaefolium* [1, 2, 3, 4, 5, 6, 7, 8] and *A. chaparense* [9]. *A. inulaefolium* is the most studied species and is one of the plants most empirically used as a medicine in the rural areas of the Colombian Andes [10]. In South America, it is used as a contraceptive in folk medicine and has been reported for use in Paraguay [3, 11].

Labdane‐type diterpenes are the main specialized metabolites described from *Austroeupatorium*. They are characterized by a 6/6 fused bicyclic carbon skeleton featuring a C‐11‐C‐16 side chain at C‐9, along with four methyl groups: two at C‐4, one at C‐8, and one at C‐10. These diterpenoids are biosynthesized from the intermediate copalyl diphosphate (CPP) or its enantiomer *ent*‐CPP, which give rise to the labdane and *ent*‐labdane backbones, respectively [12, 13]. *Ent*‐labdanes are the most commonly occurring type in *Austroeupatorium* genus [3, 4, 5, 8, 9, 14].

As part of our ongoing research on Eupatorieae species from the Campos Gerais region of Paraná, Brazil, this study focuses on *Austroeupatorium laetevirens* (Hook. & Arn.) R.M.King & H. Rob. (basionym: *Eupatorium laetevirens* Hook. & Arn.) [15]. This sub‐shrub grows up to 1 m in height, features white flowers, and is distributed across Paraguay, Argentina, and the south and southeast of Brazil [15, 16].

In this study, a molecular networking strategy was employed to facilitate the identification of labdanes in *A. laetevirens*. This approach allowed the annotation of nine labdanes, two of which were successfully isolated. One of these compounds had been previously reported in the literature from *Gutierrezia dracunculoides*; however, its structural elucidation was based only on their ^1^H NMR spectral data [17]. The other compound is described here for the first time. Therefore, this study provides a complete assignment of the hydrogens and carbons, as well as the absolute configuration of the isolated labdanes. Seven other known compounds were isolated from *A. laetevirens*. The anti‐*Mycobacterium tuberculosis* and antiproliferative potential were also assessed.

## 2 Results and Discussion

### 2.1 Molecular Networking‐Based Labdane Screening

A crude extract of the aerial parts of *A. laetevirens* was subjected to partition into hexane, dichloromethane, and ethyl acetate. With exception of hexane fraction, the other fractions were analyzed by ultra‐high performance liquid chromatograph (UHPLC)‐MS/MS quadrupole time‐of‐flight (Q‐TOF) in both positive and negative ionization modes. For the positive mode, the obtained fragmentation data were processed to generate a molecular networking through the Global Natural Products Social Networking (GNPS) platform [18]. The molecular networking for the fractions was generated from 357 parent ions. On the basis of cosine similarity, 24 clusters were formed in the annotated network. Each cluster represents a molecular family, constituted of nodes colored according to the proportion of the compound found in each fraction (Figure S2).

**FIGURE 1:**
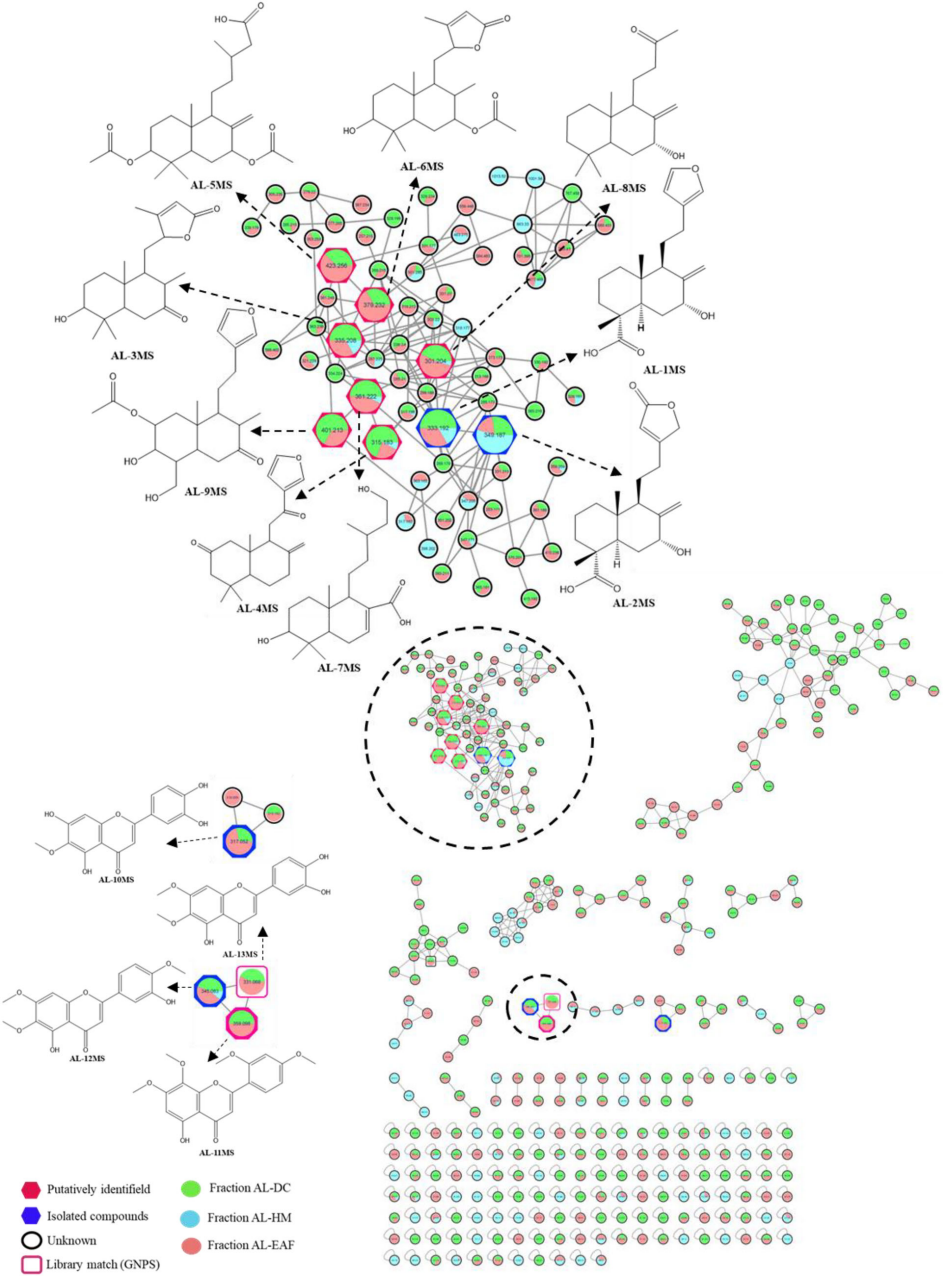
Molecular networking of the dichloromethane, ethyl acetate, and hydromethanol fractions of *Austroeupatorium laetevirens* analyzed by UHPLC–HRMS in positive mode with the expanded diterpenes of labdane‐type cluster. Nodes represent detected compounds and are colored according to the respective fraction.

A dereplication strategy based on molecular networking was conducted to maximize the speed of identifying compounds with specific structural features. The strategy was performed with the support of an established in‐house compound library based on labdane‐type diterpenes described in the literature for the genera *Austroeupatorium* and *Eupatorium*. Among the clusters formed, we highlighted the cluster shown in Figure 1, due to the presence of matches with the labdanes from our in‐house library [1, 3, 19, 20, 21, 22], which allowed the putative identification of nine labdanes (**AL‐1MS** to **AL‐9‐MS**) (Table S1). With exception of **AL‐4MS**, all the labdanes are being described for the first time in this genus. The fragmentation spectra of these compounds are shown in Figures S3–S11.

### 2.2 Isolation and Structure Elucidation

As observed through dereplication strategy using molecular networking, labdanes were present in the dichloromethane and acetate fractions; therefore, these fractions were subjected to chromatographic separations, resulting in the isolation of nine compounds. The structures of the known isolates were identified on the basis of their reported spectroscopic data. Compounds **1**–**4** were identified as the pentacyclic triterpenes: lupeol acetate (**1**) [23], α‐amyrin acetate (**2**) [24], α‐amyrin (**3**) [25], and β‐amyrin (**4**) [24]. Compounds **5** and **6** were identified as labdane‐type diterpenes, with carbon‐13 spectroscopic data from **5** being described for the first time, whereas compound **6** was identified as a new derivative. Compounds **7**–**9** were identified as the flavonoids: eupatorin (**7**) [26], eupafolin (**8**) [27], and rutin (**9**) [28] (Figure S1). Lupeol acetate (**1**), β‐amyrin (**4**), and eupafolin (**8**) were earlier reported in *Austroeupatorium* genus [3, 5, 9].

Compound **5** was isolated from the dichloromethane fraction as a white powder. The molecular formula was determined as C_20_H_28_O_4_ based on the protonated molecular ion at *m/z* 333.2050 [M + H]^+^ (calcd. for C_20_H_28_O_4_ *m/z* 333.2060; error = 3 ppm) in the HR‐ESI‐MS spectra (Figure S3). This compound had been previously described in the literature; however, the structure elucidation was based only on their ^1^H NMR spectra data [17]. The ^1^H NMR data of **5** (Figure S12), summarized in Table 1, revealed the presence of a furan ring at *δ*_H_ 6.26 (d, *J = *1.15 Hz), 7.35 (t, *J = *1.55 Hz), and 7.21 (*br. s*); an oxymethine proton at *δ*_H_ 4.43 (s); two olefinic protons at *δ*_H_ 5.11 (s) and 4.73 (s); and two singlet methyl groups at *δ*_H_ 1.13 and 0.70.

**TABLE 1:** ^1^H (300 MHz) and ^13^C (75 MHz) NMR data of compounds **5** and **6** in CDCl_3_ (*δ* in ppm, *J* in Hz).

5			6	
No.	*δ*c	*δ*_H_	*δ*c	*δ*_H_
1	37.6	1.81 (2H, m)	37.8	1.81 (2H, m)
2	18.3	1.64 (2H, m)	18.3	1.65 (2H, m)
3	33.0	1.71 (m)	33.3	1.72 (m)
		1.55 (m)		1.55 (m)
4	39.0	—	39.2	—
5	42.1	2.42 (1H, dd, *J = *2.7, 12.9)	42.2	2.47 (1H, dd, *J = *2.7, 12.9)
6	36.7	1.70 (1H, ddd, *J = *12.9, 2.7, 14.2)	36.7	1.69 (m)
				1.64 (m)
		1.52 (1H, td, *J = *2.86, 2.7, 14.0)		
7	73.7	4.43 (1H, t, *J = *2.7 and 2.7)	73.4	4.41 (1H, t, *J = *2.7 and 2.7)
8	148.2	—	147.9	—
9	50.2	2.24 (m)	50.2	2.30 (1H, m)
10	46.8	—	46.7	—
11	23.1	2.27 (m)	20.7	1.84 (2H, m)
12	23.5	2.56 (m)	26.9	2.56 (2H, m)
13	125.2	—	170.8	—
14	110.8	6.27 (1H, d, *J = *1.15)	115.2	5.87 (1H, m)
15	142.7	7.35 (1H, t, *J = *1.55)	174.2	—
16	138.6	7.21 (1H, s)	73.2	4.73 (2H, d, *J =* 1.70 Hz)
17	110.5	5.11 (1H, s)	110.2	5.12 (1H, s)
		4.73 (1H, s)		4.62 (1H, s)
18	16.2	1.13 (3H, s)	16.2	1.14 (3H, s)
19	182.9	—	182.3	—
20	13.7	0.70 (3H, s)	13.6	0.73 (3H, s)

**FIGURE 2:**
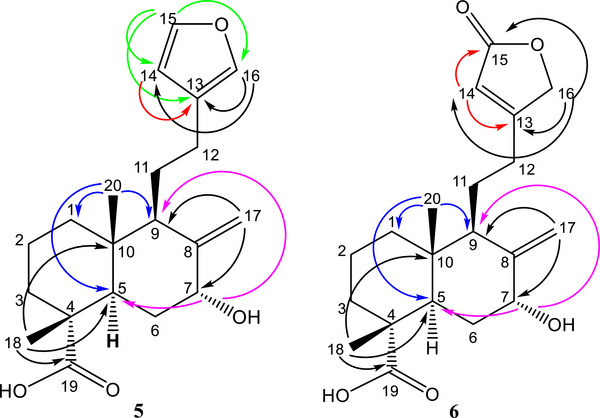
Main correlations observed in the HMBC spectrum for the compounds **5** and **6**.

**FIGURE 3:**
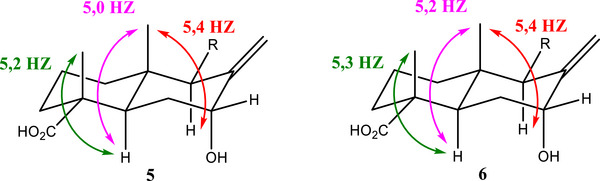
Figure showing the correlations and bracket constants according to the pip‐HSQMBC spectrum for determining the relative configuration of compounds **5** and **6**.

Analysis of the HSQC data revealed 22 carbon resonances in the ^13^C NMR spectrum (Figures S13 and S14) and confirmed the presence of a furan ring at *δ*_C_ 125.2 (C‐13), 110.8 (C‐14), 142.7 (C‐15), 138.6 (C‐16); a carbonyl group at *δ*_C_ 182.9 (C‐19); an exocyclic methylene at *δ*_C_ 110.5 (C‐17); and methyl groups at *δ*_C_ 16.2 (CH_3_‐18) and *δ*_C_ 13.7 (CH_3_‐20). The connectivity and confirmation of the structure were obtained from the HMBC spectrum (Figure S15), as shown in Figure 2. The spectrum displayed correlations between the hydrogen at *δ*_H_ 7.35 (H‐15) with the carbons *δ*_C_ 138.6 (C‐16), 125.2 (C‐13), and 110.8 (C‐14), the hydrogen at *δ*_H_ 7.21 (H‐16) with the carbons *δ*_C_ 142.7 (C‐15), 125.2 (C‐13), and 110.8 (C‐14) as well as the hydrogen at *δ*_H_ 6.27 (H‐14) with *δ*_C_ 125.2 (C‐13), 138.6 (C‐16), and 142.7 (C‐15) confirming the furan ring. In addition, correlations between hydrogens at *δ*_H_ 5.11 (s) and 4.73 (s) with the carbons *δ*_C_ 50.2 (C‐9) and 73.7 (C‐7) were observed. The oxymethine proton at *δ*_H_ 4.43 (s) showed correlations with the carbons at *δ*_C_ 42.1 (C‐5), 50.2 (C‐9), and 110.5 (C‐17). The connectivity of the methyl groups was confirmed due to the correlations of *δ*_H_ 0.70 (H‐20) with the carbons *δ*_C_ 37.6 (C‐1), 42.1 (C‐5), and 50.2 (C‐9) and of *δ*_H_ 1.13 (H‐18) with the carbons at *δ*_C_ 42.1 (C‐5), 46.8 (C‐10), and with the carboxylic acid at *δ*_C_ 182.9 (C‐19). The relative configuration of compound **5** was determined by analyzing the ^3^ *J*_CH_ coupling constants obtained from pip‐HSQMBC‐TOCSY‐IPAP [29] spectra for carbons C4, C9, and C10 (Figures S16 and S17). The analysis was carried out using a Bruker Avance III HD spectrometer operating at 500 MHz for the ^1^H, with a 1.7 mm prob., and the sample diluted in CDCl_3_. ^3^ *J*_CH_ constants were selected for analysis due to their behavior analogous to ^3^ *J*_HH_ with respect to dihedral angles, as described by the Karplus curve. Typically, these constants range from 0 to 9 Hz, with the highest values observed when the C─C─C─H dihedral angle adopts an anti‐configuration. The axial position of H5 served as a reliable reference point for determining the relative configurations of C4 and C10. The methyl groups attached to C4 and C10 can adopt either anti‐ or gauche orientations relative to H5. For C4, the ^3^ *J*_CH_ constant between H5 and the methyl carbon (C18) was measured at 5.2 Hz. For C10, the ^3^ *J*_CH_ constant between H5 and the methyl carbon (C20) was 5.0 Hz. Both high values, close to 6 Hz, indicate that these methyl groups are in an anti‐position to H5. As H5 had already been identified in the axial position of the fused ring, the methyl groups also occupy axial positions, thereby defining the relative configurations of C4 and C10. To assess the configuration at C9, the ^3^ *J*_C20‐H9_ coupling constant was analyzed, yielding a value of 5.4 Hz. This indicates that both H9 and the methyl group (C20) are in axial positions. Consequently, the relative stereochemistry is consistent with a *trans*‐decalin derivative configuration for the bicyclic system, as illustrated in Figure 3.

**FIGURE 4:**
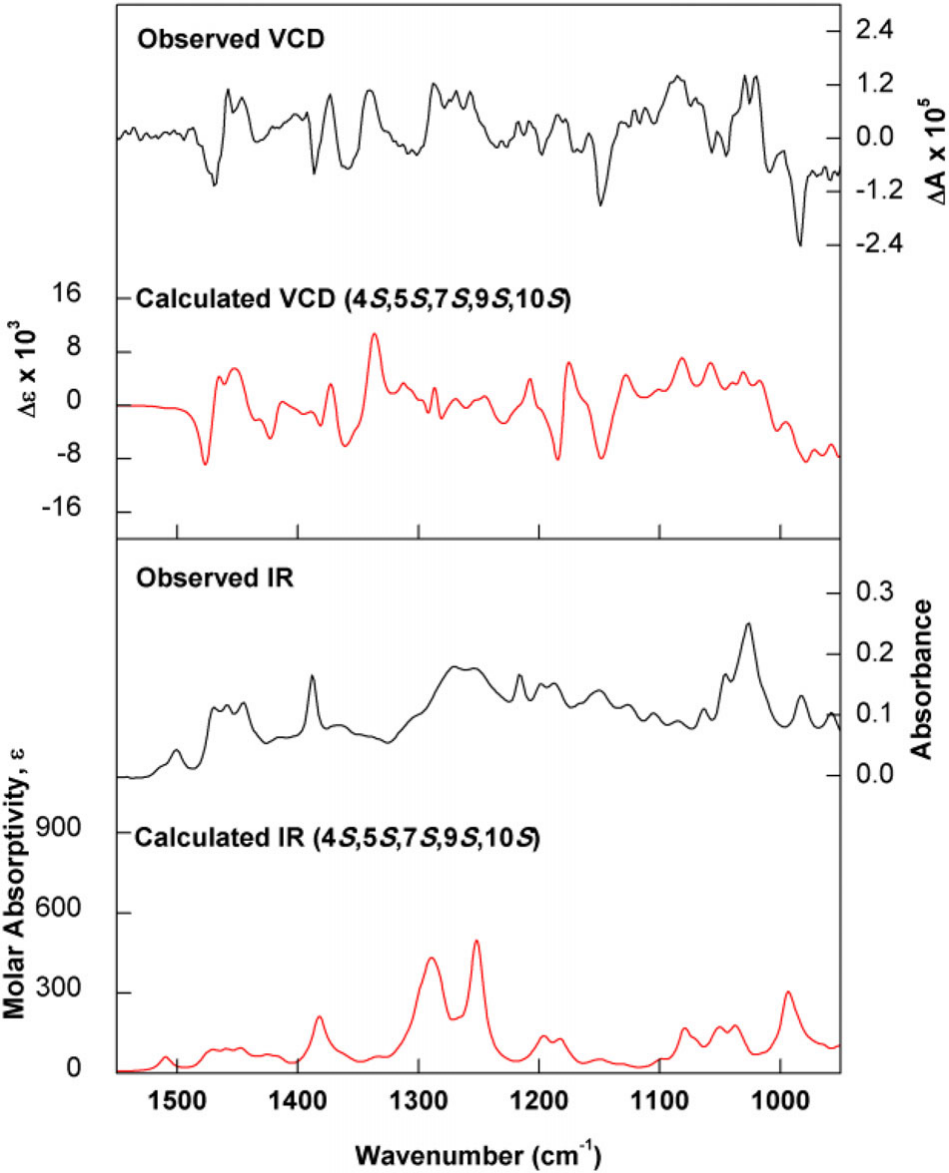
Comparison between experimental IR and VCD spectra of (−)‐5 (black trace) with calculated [B3PW91/PCM(CHCl_3_)/6‐311G(d,p)] data for (4*S*,5*S*,7*S*,9*S*,10*S*)‐5 as a carboxylic acid dimer (red trace). See Supporting Information section for optimized structures and energies of the lower energy conformers.

After establishing the relative configuration of compound **5**, comparisons of experimental infrared (IR) and vibrational circular dichroism (VCD) data with density functional theory (DFT) simulated spectra were performed to determine its absolute configuration. As the initial agreement between experiment and theory was not satisfactory, especially in the IR, simulations of carboxylic acid dimers with formic acid were performed. This procedure has been demonstrated to significantly improve the reproduction of experimental IR and VCD of carboxylic acids in chloroform solution [30]. The good agreement between observed and calculated (Figure 4) data allowed the assignment of (−)‐**5** as 4*S*,5*S*,7*S*,9*S*,10*S*.

Compound **6** was isolated from the dichloromethane fraction as a white powder. The molecular formula was determined as C_20_H_28_O_5_ on the basis of the protonated molecular ion at *m/z* 349.1996 [M + H]^+^ (calcd. for C_20_H_28_O_5_ *m/z* 349.2010; error* = *4.01 ppm) in the HR–ESI–MS spectra (Figure S4). The ^1^H NMR (Figure S18) data of compound **6** (Table 1) displayed characteristic resonances indicating an α,β‐unsaturated *γ*‐lactone moiety with two protons at *δ*_H_ 4.73 (d, *J = *1.70) and 5.87 (m), an oxymethine proton at *δ*_H_ 4.41 (s), two olefinic protons at *δ*_H_ 5.12 (s) and 4.62 (s), and two singlet methyls at *δ*_H_ 1.14 and 0.73. Analysis of the HSQC data revealed 22 carbon resonances in the ^13^C NMR spectrum (Figures S19 and S20) and confirmed the presence of an α,β‐unsaturated *γ*‐lactone moiety (Table 1) due to the signals at *δ*_C_ 170.8 (C‐13), 115.2 (C‐14), 174.2 (C‐15), and 73.20 (C‐16). The ^13^C NMR spectrum also exhibited signals for a carbonyl group at *δ*_C_ 182.3 (C‐19), an exocyclic methylene at *δ*_C_ 110.2 (C‐17), and methyl groups at *δ*_C_ 16.2 (CH_3_‐18) and *δ*_C_ 13.6 (CH_3_‐20). In addition, signals for the decaline system were assigned to four methylene groups at *δ*_C_ 37.8 (C‐1), 18.3 (C‐2), 33.3 (C‐3), and 36.7 (C‐6); three methines at 42.2 (C‐5), 73.4 (C‐7), and 50.2 (C‐9); and three quaternary carbons at 39.2 (C‐4), 147.9 (C‐8), and 46.7 (C‐10). Apart from the signals of the α,β‐unsaturated *γ*‐lactone moiety, the NMR characteristics of the compound were similar to those of compound **5**.

The connectivity and structure confirmation of compound **6** were obtained from the HMBC spectrum (Figure S21). The spectrum displayed correlations between the hydrogen at *δ*_H_ 5.87 (H‐14) with the carbons at *δ*_C_ 73.2 (C‐16), 174.5 (C‐15), and 170.8 (C‐13), as well as between the hydrogen at *δ*_H_ 4.73 (H‐16) and the carbons at *δ*_C_ 115.2 (C‐14), 174.5 (C‐15), and 170.8 (C‐13), confirming the presence and position of the α,β‐unsaturated *γ*‐lactone. The two olefinic protons at *δ*_H_ 5.12 (s) and 4.62 (s) showed correlations with the carbons at *δ*_C_ 50.2 (C‐9) and 73.4 (C‐7), as in compound **5**. The oxymethine proton at *δ*_H_ 4.41 (s) showed correlations with the carbons at *δ*_C_ 42.2 (C‐5), 50.2 (C‐9), and 110.2 (C‐17). The presence and position of the methyl group were confirmed due to the correlations of *δ*_H_ 0.73 (H‐20) with the carbons at *δ*_C_ 37.8 (C‐1), 42.2 (C‐5), and 50.2 (C‐9), and of *δ*_H_ 1.14 (H‐18) with the carbons at *δ*_C_ 42.2 (C‐5), 46.7 (C‐10), and with the carboxylic acid at *δ*_C_ 182.3 (C‐19). These correlations are shown in Figure 2.

The relative configurations of C4, C10, and C9 were determined by analyzing the ^3^ *J*_CH_ coupling constants obtained in pip‐HSQMBC‐TOCSY‐IPAP spectra (Figure S22), as previously performed for compound **5**. To determine the configuration of C4, the ^3^ *J*_CH_ constant between H5 and the methyl carbon C18 was analyzed, yielding a value of 5.3 Hz. For C10, the ^3^ *J*_CH_ constant between H5 and the methyl carbon C20 was found to be 5.2 Hz. Both values indicate that the methyl groups are in an anti‐position relative to H5. This establishes the relative configuration of C4 and C10, confirming that the analyzed bicyclic compound adopts a *trans*‐decalin derivative configuration. For C9, the ^3^ *J*_C20‐H9_ coupling was analyzed, yielding value of 5.4 Hz, consistent with the earlier observations. This indicates that both H5 and the methyl group C20 are in axial positions, defining the relative stereochemistry as illustrated in Figure 3.

**FIGURE 5:**
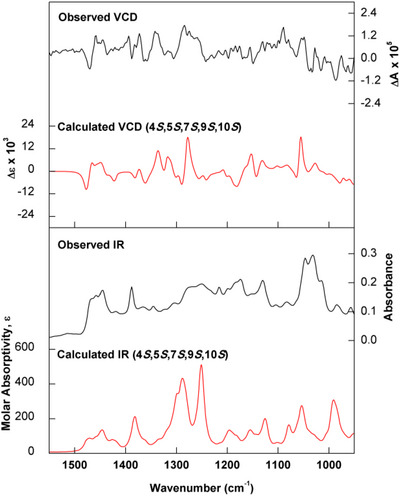
Comparison between experimental IR and VCD spectra of (−)‐**6** (black trace) with calculated [B3PW91/PCM(CHCl_3_)/6‐311G(d,p)] data for (4*S*,5*S*,7*S*,9*S*,10*S*)‐**6** as a carboxylic acid dimer (red trace). See Supporting Information section for optimized structures and energies of the lower energy conformers.

The absolute configuration of compound **6** was determined using the same approach as for compound **5**. The experimental IR and VCD data of (−)‐**6** were compared to simulated spectra obtained through DFT simulations of a carboxylic acid dimer. The good agreement between the observed and calculated data (Figure 5) allowed the assignment of the absolute configuration of (−)‐**6** as 4*S*,5*S*,7*S*,9*S*,10*S*.

### 2.3 Molecular Networking and Dereplication

To obtain the annotation of other compounds in positive ionization mode, compound **7**, the flavonoid eupatorin, which was isolated and characterized by NMR analysis and identified by UHPLC–high‐resolution mass spectrometer (HRMS)/MS as AL‐12MS, was used as a standard. Its isolation allowed its detection in the molecular network, facilitating the identification of other three flavonoids (Table S2) (Figure 1). The substances were putatively identified by the GNPS platform databases, and through comparison with the literature, AL‐11MS is being described for the first time in the genus [31, 32, 33, 34].

To annotate the compounds that did not form clusters, the crude extract and the fractions were analyzed in the negative ionization mode (Table S3). This led to the putative identification of 40 compounds. The data obtained for the negative ionization mode were putatively identified according to the fragmentation profiles of the spectra generated in comparison with literature data, mainly with substances isolated from the Asteraceae family and available databases (HMDB, MoNA, MassBANK.EU, FooDB).

### 2.4 Activity

#### 2.4.1 Antiproliferative Assay

The antiproliferative assay was evaluated using the MTT assay. For an initial screening of the cytotoxic potential exerted by the crude extract of *A. laetevirens*, fractions were assayed against cells of the HCT‐116 (colorectal carcinoma) at two concentrations (50 and 5 µg). However, the data obtained showed that the extracts and fractions did not exhibit any significant activity against the HCT‐116 cell line (Table S4).

#### 2.4.2 Anti‐*Mycobacterium tuberculosis* Activity

The in vitro anti*‐M. tuberculosis* activity of ethanolic crude extract, the hexane, dichloromethane, ethyl acetate, and hydrometanol fractions, and the isolated compounds of *A. laetevirens* aerial parts was evaluated using Resazurin Microtiter Assay Plate (REMA). The ethyl acetate fraction showed weak activity with minimum inhibitory concentration (MIC) value of 250 µg mL^−1^, whereas the flavonoids eupatorin (**7**) and eupafolin (**8**) demonstrated moderate activity against *M. tuberculosis* H_37_Rv ATCC 27294, with MIC values of 125 µg mL^−1^ (Table S5).

## 3 Conclusion

In this study, a molecular networking approach facilitated the identification of labdanes from *A. laetevirens*. Two of these labdanes were isolated for the first time, and their absolute configurations were determined using NMR, VCD, and DFT calculations. This network‐based approach allowed us to explore intricate connections between various metabolites, leading to the identification of an additional 25 compounds. The flavonoids eupatorin and eupafolin exhibited a moderate antimycobacterial activity, with MIC values of 125 µg mL^−1^ against *M. tuberculosis* H_37_Rv. Despite the lack of significant antiproliferative effects against the HCT‐116 cell line, these findings contribute valuable chemical insights and enrich the scaffolds of diterpenes in the genus.

## 4 Experimental Section

### 4.1 General Experimental Procedures

Separations by column chromatography (CC) were carried out using silica gel 60 (70–230 mesh, Merck), silica gel flash (230–400 mesh, Acros Organics), or Sephadex LH‐20 (Sigma). Thin layer chromatography (TLC) was performed on normal phase pre‐coated silica gel 60G or 60GF_254_ (Merck) plates. The compounds on TLC were visualized by UV irradiation at 254 and 366 nm and/or by spraying with an H_2_SO_4_/anisaldehyde/acetic acid (1: 0.5: 50 mL) solution followed by heating at 100°C. NMR spectra were recorded in CDCl_3_ and dimethyl sulfoxide (DMSO)‐*d*_6_ on a Bruker Avance III HD spectrometer operating at 300 and 75.5 MHz. Optical rotations were measured at 24°C on a PerkinElmer Model 343 polarimeter. High‐resolution mass spectra were obtained in a QTOF, Bruker Daltonics, model Impact II spectrometer in electrospray ionization. UHPLC separation was performed using C18 columns (75 × 2.0 mm^2^ i.d.; 1.6 µm Shim‐pack XR‐ODS III) on a Shimadzu, model Nexera X2. IR and VCD experimental spectra were recorded simultaneously with a BioTools dual‐photoelastic modulator (PEM) Chiral*IR*‐2X FT‐VCD spectrometer using a resolution of 4 cm^−1^ and a collection time of 15 h. The optimum retardation of the ZnSe PEMs was set at 1400 cm^−1^. The IR and VCD spectra of **5** and **6** were recorded in chloroform‐*d*_1_ solution (9.0 mg in 150 µL) in a BaF_2_ cell with 100 µm path length. Minor instrumental baseline offsets were eliminated by subtracting the VCD spectra of the solvent recorded under identical conditions.

### 4.2 Plant Materials

The aerial parts of *A. laetevirens* were collected in Ponta Grossa city, Paraná State, Brazil (25°12′10″ S; 49°56′37″ W) in September 2019 and identified by Dra. Marta Regina Barrotto do Carmo. A voucher specimen (HUPG 22505) was deposited at the herbarium at Universidade Estadual de Ponta Grossa (SISGEN AB3C23F).

### 4.3 Analysis of the Fractions by UHPLC–HRMS/MS

The fractions were analyzed using an UHPLC coupled to a high‐resolution mass spectrometer (UHPLC–HRMS/MS) according to method described by Araujoet al. [35]. The samples were prepared in CH_3_OH (2.0 mg mL^−1^), filtered through a 0.22 µm filter, and chromatographic separations were performed using UHPLC on a Symmetry C18 column (75 × 2.0 mm^2^ i.d.; 1.6 µm Shim‐pack XR‐ODS III) maintained at 40°C. The mobile phase consisted of H_2_O (solvent A) and 0.1% formic acid in CH_3_OH (solvent B). The gradient program was as follows: initial 0–1 min, using elution A–B (95:5, v/v), 1–3 min (30:70 v/v), 3–12 min (5:95 v/v), and maintained at (5:95 v/v) until 16 min, at a flow rate of 0.2 mL min^−1^ and an injection volume of 3 µL. High‐resolution mass spectrometry analysis was carried out on a Q‐TOF mass spectrometer with an electrospray ionization interface. The capillary voltage was set at 4500 V for positive and negative ionization modes, using sodium formate (10 µM) as calibrant. The dry gas parameters were set to 8 L min^−1^ at 200°C with a nebulization gas pressure of 4 bar. Collision‐induced dissociation (CID) fragmentation was performed using nitrogen (N_2_) collision gas and collision energy from 15 to 30 eV. Spectra data were collected from *m/z* 50–1300, with an acquisition rate of 5 spectra per second. The ions of interest were selected by auto MS/MS scan fragmentation. The data processing software was Data Analysis 4.3 (Bruker), and putative identification of substances in the extracts and fractions was performed on the basis of the fragmentation profiles of the spectra generated and compared with literature data of compounds previously identified in the genus *Austroeupatorium*, as well as databases such as MassBank (http://www.massbank.jp/), Human Metabolome Database (http://www.hmdb.ca/), and Food Database (http://foodb.ca/). Moreover, a mass error value was calculated, and molecular formulas with a mass error value of ≤10 ppm were considered in this study [36].

### 4.4 Molecular Networking

The raw data from the analyses performed using UHPLC–MS/MS were converted to mzXML format using Bruker data software. The resulting data were then uploaded to GNPS, and a molecular network was generated using the online workflow on the GNPS website. The data were filtered by removing all MS/MS fragment ions within +/−17 Da of the precursor *m/z*. MS/MS spectra were window filtered by selecting only the top six fragment ions in the +/−50 Da window throughout the spectrum. The precursor ion mass tolerance was set to 0.02 Da and the MS/MS fragment ion tolerance of 0.02 Da. A network was created with edges filtered to have a cosine score above 0.7 and more than four matched peaks. The spectra in the network were then compared against GNPS’ spectral libraries. The library spectra were filtered in the same manner as the input data. Matches between network spectra and library spectra were kept only if they had a score above 0.7 and at least four matched peaks. The resulting network analysis was exported from GNPS and analyzed in Cytoscape [18, 37]. In addition, the obtained data were also compared with data from the literature to further aid in compound identification.

### 4.5 Extraction and Isolation

The air‐dried powder of aerial parts of *A. laetevirens* (129 g) was extracted with ethanol (5 × 0.5 L) at room temperature, and the solvent evaporated under vacuum. The resulting ethanol extract (AL‐CE, 19 g) was dissolved in methanol:water (50:50) and partitioned into hexane, dichloromethane, and ethyl acetate. The solvents were evaporated, resulting in the hexane (AL‐HEX, 3.35 g), dichloromethane (AL‐DC, 5.85 g), ethyl acetate (AL‐EAF, 2.80 g), and residual hydromethanol (AL‐HM, 4.01 g) fractions.

The hexane fraction was subjected to silica gel column using a gradient solvent system of hexane/EtOAc/methanol to afford the subfractions AL‐HEX.1 to AL‐HEX.20. The subfraction AL‐HEX.2 afforded a mixture of compounds **1** and **2** (1.07 g), whereas subfraction AL‐HEX.4 afforded a mixture of compounds **1**, **3**, and **4** (105 mg).

The dichloromethane fraction was subjected to silica gel CC using a gradient solvent system of hexane/EtOAc/methanol to afford the subfractions AL‐DC.1 to AL‐DC.18. Subfraction AL‐DC.1 afforded compounds **1** and **2** (163 mg) in a mixture, the subfraction AL‐DC.10 afforded compound **5** (355 mg), and subfraction AL‐DC.11 afforded compound **7** (7.8 mg). Compound **6** (84 mg) was obtained by purifying subfraction AL‐DC.16 (1.09 g) on silica gel flash CC using a mixture of CHCl_3_ and methanol as eluent.

The ethyl acetate fraction was purified using Sephadex LH‐20 eluted with methanol/water mixture, resulting in subfractions AL‐EAF.1 to AL‐EAF.10. Compound **8** (11 mg) was obtained from subfraction AL‐EAF.8. Similarly, the hydromethanol fraction was subjected to purification in Sephadex LH‐20 using methanol/water to afford the subfractions AL‐HM.1 to AL‐HM.17. The subfraction AL‐HM.12 afforded compound **9** (11 mg).

### 4.6 Calculations

The conformational searches of compounds **5** and **6** were carried out at the molecular mechanics level of theory employing both the MM+ and MMFF force fields incorporated in HyperChem 8.0.10 and Spartan 08 software packages, respectively. The DFT calculations were carried out at 298 K in chloroform solution using the polarizable continuum model (PCM) in its integral equation formalism version (IEFPCM), incorporated in Gaussian 09 software [38]. The configuration 4*R*,5*R*,7*R*,9*R*,10*R* was arbitrarily chosen for **5** and **6**, based on the relative configuration determined by NMR. The VCD properties of their enantiomers were obtained by multiplying the calculated data by (−1). Initially, 100 conformers were identified for **5** and **6** within a 10 kcal mol^−1^ energy window. These conformers were then geometry optimized at the B3PW91/PCM(CHCl_3_)/6‐311G(d,p) level. The 9 and 13 lowest energy conformers of **5** and **3**, respectively, with relative energy (rel E.) ≤2.0 kcal mol^−1^ were selected for IR/VCD calculations. IR and VCD were simulated at the B3PW91/PCM(CHCl_3_)/6‐311G(d,p) level, and the spectra were created using dipole and rotational strengths from Gaussian, which were converted into molar absorptivity (M^−1^ cm^−1^). Each spectrum was plotted as a sum of Lorentzian bands with half‐widths at half‐maximum (HWHM) of 6 cm^−1^. The calculated wavenumbers were multiplied with a scaling factor of 0.98, and the simple‐average‐composite IR and VCD spectra were plotted using Origin software. Compounds **5** and **6** were further investigated as dimers with formic acid to simulate the dimerization of carboxylic acids observed in chloroform solution. The molecular complexes (dimers) were reoptimized and had their IR and VCD spectra calculated at the same level as the isolated molecules [B3PW91/PCM(CHCl_3_)/6‐311G(d,p)].

### 4.7 Activity

#### 4.7.1 Antiproliferative Assay

Cytotoxicity of ethanolic crude extract, hexane, dichloromethane, ethyl acetate, and hydromethanol fractions of *A. laetevirens* aerial parts was evaluated by the MTT assay [39] against the human tumor cell line HCT‐116 (colorectal carcinoma). Cells were plated in 96‐well plates (10^4^ cells well^−1^ in 200 µL well^−1^) and left to adhere for 24 h. Each sample, dissolved in DMSO, was added to their respective wells, making up to final concentrations of 5 and 50 µg mL^−1^. Serial dilution of doxorubicin was used as a positive control, whereas negative control groups received vehicle. After 72 h of incubation, the supernatant was substituted with fresh media added with 0.5 mg mL^−1^ of MTT (3‐(4,5‐dimethyl‐2‐thiazolyl)‐2,5‐diphenyl‐2*H*‐tetrazolium bromide) (Sigma‐Aldrich, USA). After 3 h, the supernatant was removed, and the plates were left to dry in a warm house for 30 min. The precipitate products were dissolved in 150 µL DMSO, and the absorbance was measured using a multiplate reader at 570 nm. The percentage of cell growth inhibition for each sample was determined by analysis in GraphPad Prism v8.0 software.

#### 4.7.2 Anti‐*Mycobacterium tuberculosis* Activity Assay

The anti‐*M. tuberculosis* activity of the extracts and compounds was determined using the Resazurin Microtiter Assay Plate REMA method according to Palomino et al. [40] for determination of the MIC. The reference strain *M. tuberculosis* H_37_Rv (ATCC 27294) was cultured in Middlebrook 7H9 broth medium (Difco Laboratories, USA), supplemented with 10% of oleic acid, bovine albumin, dextrose, and catalase (OADC) enrichment (7H9‐OADC) (BBL/Becton Dickinson, USA), and 0.2% of glycerol (Sigma‐Aldrich, USA), for 15–21 days at 35°C. Stock solutions of the tested extract, fraction, and compounds were prepared in DMSO. In 96‐well plate, 100 µL of 7H9‐OADC was added to all wells of columns 2–11. In the second well, 100 µL of samples were added, and then a serial dilution carried out to column 10, in which the concentrations ranged from 250 to 0.98 µg mL^−1^. Then, it was added 100 µL of the diluted *M. tuberculosis* H_37_Rv in all wells in columns 2–10, and 4 wells of column 11 (positive control), the other 4 wells were the negative control. Isoniazid was used as the reference drug at concentrations from 0.007 to 1.0 µg mL^−1^. The plate was sealed and incubated for 7 days at 37°C. After that time, 30 µL of a freshly prepared 0.02% resazurin solution (Sigma‐Aldrich, USA) was added, and the microplate incubated for 24 h. A color change from blue (oxidized state) to pink (reduced) indicated bacterial growth and MIC was defined as the lowest drug concentration that prevented the color change.

## Author Contributions

**Francielli A. P. Valeze**: performed the phytochemical studies and identified the isolated compounds, analyzed the data obtained from UHPLC–HRMS/MS with the molecular networking strategy. **Maria Eduarda V. de Souza**: performed the phytochemical studies and identified the isolated compounds. **Beatriz P. Moreno**: analyzed the data obtained from UHPLC–HRMS/MS with the molecular networking strategy. pip‐HSQMBC‐TOCSY‐IPAP analysis was carried on by **Cleverton S. Fernandes** and **Ernani A. Basso**. Absolute configuration was determined by Andrea **N. L. Batista** and **João M. Batista Junior**. Biological assays were conducted and analyzed by **Bianca D. B. Sahm**, **Andressa L. Ieque**, **Regiane B. L. Scodro**, **Leticia V. Costa‐Lotufo**. **Marta R. B. do Carmo** collected and identified the aerial parts of the species. **Maria H. Sarragiotto** conceived the present idea, developed the method, and supervised the findings of this work. **Debora C. Baldoqui** conceived the present idea, developed the method, and supervised the findings of this work.

## Conflicts of Interest

The authors declare no conflicts of interest.

## Supporting information




**Supporting File 1**: cbdv70540‐sup‐0001‐SuppMat.pdf

## Data Availability

The data that support the findings of this study are available in the Supporting Information of this article.
