# Supplementary material for: Bioprospecting of Labdane‐Type Diterpenes From Austroeupatorium laetevirens: Molecular Networking, Structural Elucidation, and Biological Activity Evaluation
Source: Chem Biodivers. 2025 Oct 10;22(12):e02381. doi: 10.1002/cbdv.202502381 (PMC12716004; doi:10.1002/cbdv.202502381)
Supplement: Supplementary file 1 — Supporting File 1: cbdv70540‐sup‐0001‐SuppMat.pdf [file CBDV-22-e02381-s001.docx]

**Supplementary Information**

**Figure 1S** - Compounds isolated from *Austroeupatorium laetevirens.*

**Figure 2S** - Molecular networking of the dichloromethane, ethyl acetate and hydromethanol fractions of *Austroeupatorium laetevirens* analyzed by UHPLC-HRMS in positive mode. Nodes represent detected compounds and are colored according to the respective fraction with green (dichloromethane), pink (ethyl acetate), and blue (hydromethanolic). The blue octagonal nodes represent the substances isolated in the present work, red octagonal nodes represent putatively identified substances, and pink square nodes represent substances identified by the GNPS platform.


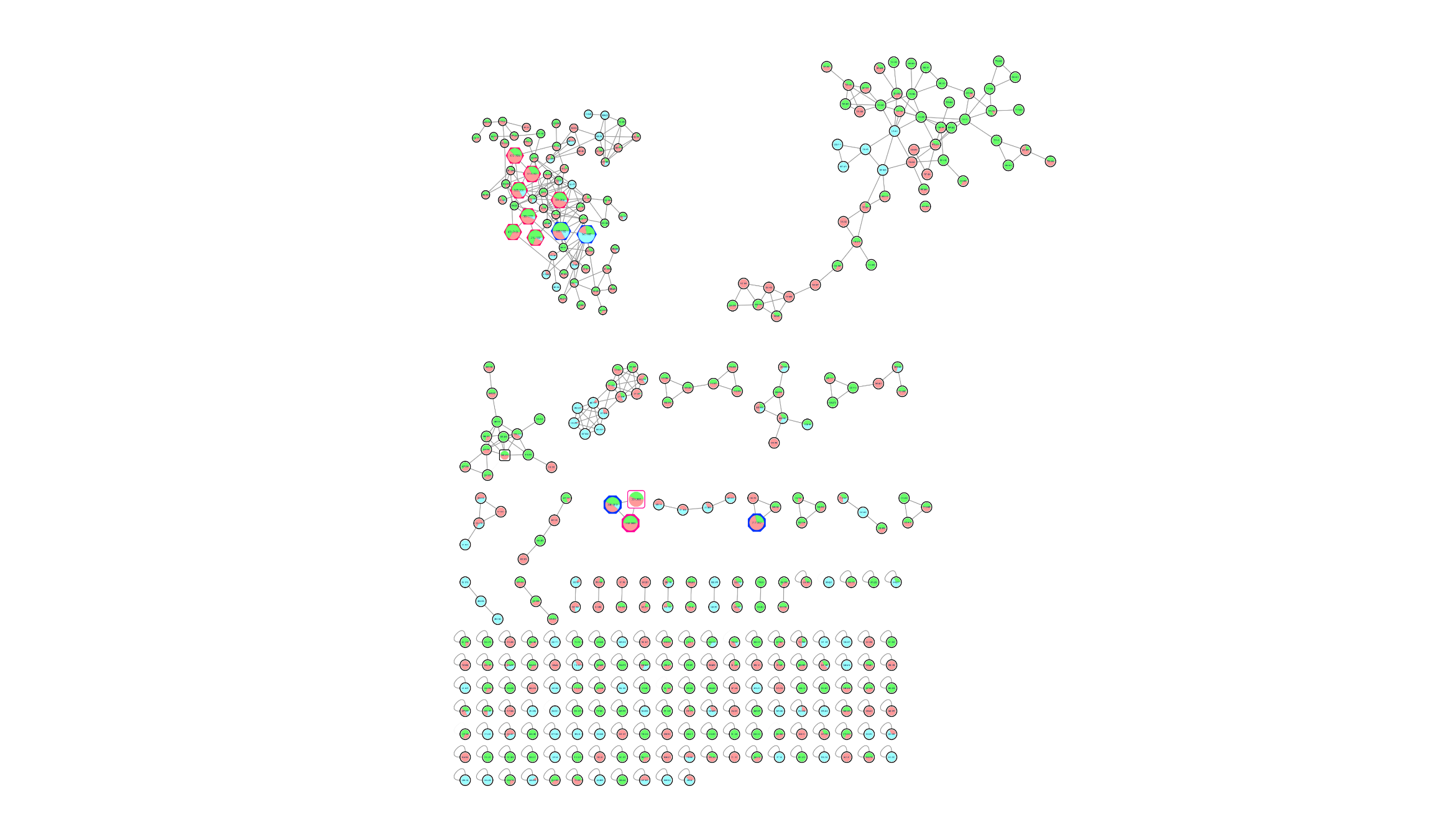


**Table 1S** - Labdanes data putatively identified in *A. laetevirens* fractions by UHPLC-HRMS/MS and *molecular networking*.

| **Compound** | **Molecular formula** | | **Exact mass (*m/z)*** | **Íon precursor (*m/z)*** | **Error / ppm** | | **Rt / min** | **Fragments** |
| --- | --- | --- | --- | --- | --- | --- | --- | --- |
| AL-1MS | | C_20_H_28_O_4_[M + H]^+^ | 333.2060 | 333.2050 | | 3.00 | 5.00 | 270; 171; 157; 145; 119; 105; 93; 81 |
| AL-2MS | | C_20_H_28_O_5_ [M + H]^+^ | 349.2010 | 349.1996 | | 4.01 | 4.70 | 283; 255; 237;187; 171; 145; 119; 105; 95; 81 |
| AL-3MS^e^ | | C_20_H_30_O_4_ [M + H]^+^ | 335.2217 | 335.2202 | | 4.47 | 5.68 | 271; 225; 183; 159; 147; 105; 93; 81 |
| AL-4MS^a^ | | C_20_H_26_O_3_ [M+ H]^+^ | 315.1955 | 315.1944 | | 3.49 | 5.76 | 254; 195; 121; 119; 105; 91; 81 |
| AL-5MS^b^ | | C_24_H_38_O_6_ [M + H]^+^ | 423.2741 | 423.2724 | | 4.01 | 5.62 | 357; 330; 285; 267; 225; 211; 199; 159; 145; 133; 119; 105; 95 |
| AL-6MS^c^ | | C_22_H_34_O_5_ [M + H]^+^ | 379.2479 | 379.2462 | | 4.48 | 4.90 | 285; 267; 255; 197; 183; 171; 145;119; 105; 93; 81 |
| AL-7MS^d^ | | C_20_H_34_O_4_ [M + Na]^+^ | 361.2355 | 361.2351 | | 1.11 | 4.79 | 328; 269; 213; 157; 145; 119; 91; 81 |
| AL-8MS^b^ | | C_18_H_30_O_2_ [M + Na]^+^ | 301.2143 | 301.2140 | | 0.99 | 5.06 | 255; 199; 159; 131; 119; 105; 91 |
| AL-9MS^f^ | | C_21_H_30_O_6_ [M + Na]^+^ | 401.1940 | 401.1906 | | 8.47 | 4.96 | 383; 343; 283; 187; 133; 119; 105; 93 |

Rt: Retention time; Reference: ^a^Bohlmann et al., (1977); ^b^González et al., 1990; ^c^Manchand et al., 1979; ^d^Zhang et al.*,* 2008; ^e^Castillo et al., 2016; ^f^Bohlmann et al., 1984).

**Table 2S –** Flavonoids data putatively identified in *A. laetevirens* fractions by UHPLC-HRMS/MS and *molecular networking*.

| **Compound** | | **Molecular formula** | **Exact mass (*m/z)*** | **Íon precursor (*m/z)*** | **Error / ppm** | **Rt / min** | **Fragments** |
| --- | --- | --- | --- | --- | --- | --- | --- |
| AL-10MS | C_16_H_12_O_7_ [M + H]^+^ | | 317.0656 | 317.0634 | 6.93 | 4.39 | 303; 302; 168 |
| AL-11MS | C_19_H_18_O_7_ [M + H]^+^ | | 359.1131 | 359.1112 | 5.29 | 5.28 | 343; 298; 162; 136 |
| AL-12MS | C_18_H_16_O_7_ [M + H]^+^ | | 345.0946 | 345.0945 | 0.28 | 4.89 | 312; 284; 269; 185; 148;108 |
| AL-13MS | C_17_H_14_O_7_ [M + H]^+^ | | 331.0812 | 331.0794 | 5.43 | 4.59 | 316; 298; 242; 213; 168; 136; 108 |

tr: tempo de retenção; Dados da literatura: ^a^Cui et al.. 2021; ^b^Luo et al.. 2019; ^c^Li et al.. 2019; ^d^ Xia et al.. 2019.

**Figure 3S -** ESI (+)-HRMS/MS (+) spectrum of compound 5 identified as AL-1MS.

**Figure 4S -** ESI (+)-HRMS/MS (+) spectrum of compound 6 identified as AL-2MS

**Figure 5S** - ESI (+)-HRMS/MS (+) spectrum of compound AL-3MS.

**Figure 6S** - ESI (+)-HRMS/MS (+) spectrum of compound AL-4MS.

**Figure 7S** - ESI (+)-HRMS/MS (+) spectrum of compound AL-5MS.

**Figure 8S** - ESI (+)-HRMS/MS (+) spectrum of compound AL-6MS.

**Figure 9S** - ESI (+)-HRMS/MS (+) spectrum of compound AL-7MS.

**Figure 10S -** ESI (+)-HRMS/MS (+) spectrum of compound AL-8MS.

**Figure 11S -** ESI (+)-HRMS/MS (+) spectrum of compound AL-9MS.

**Table 3S -** Putatively identified compounds in dichloromethane fraction of *A*. *laetevirens* by UHPLC-HRMS/MS in negative ionization mode.

| Substância | Fórmula | Massa exata | Íon precursor  [M-H]^-^ (*m/z)* | Erro / ppm | t_R_/min | Fragmentos  (*m/z)* | Fração | Identificação Putativa | Database |
| --- | --- | --- | --- | --- | --- | --- | --- | --- | --- |
| AL-14MS | C_3_H_6_O_3_ | 89.0236 | 89.0236 | 2.24 | 0.79 | - | EB. FHM | Lactic acid | MoNA |
| AL-15MS | C_6_H_12_O_7_ | 195.0504 | 195.0499 | 2.56 | 0.87 | 87; 129; 151 | EB. FAE; FHM | Gluconic acid | HMDB |
| AL-16MS | C_7_H_6_O_3_ | 137.0238 | 137.0242 | -2.91 | 4.54 | 93 | EB; FAE; FHM | *p*-Hydroxybenzoic acid | MoNA |
| AL-17MS | C_7_H_6_O_4_ | 153.0187 | 153.0189 | 1.30 | 4.19 | 91; 109 | EB; FAE; FHM | Protocatechuic acid | HMDB |
| AL-18MS | C_9_H_8_O_3_ | 163.0394 | 163.0394 | 0 | 4.91 | 93; 119 | EB; FAE | p-Coumaric acid | MoNA |
| AL-19MS | C_9_H_16_O_4_ | 187.0970 | 187.0970 | 0 | 5.11 | 97; 125; 143; 169 | FDC; FAE | Azelaic acid | HMDB |
| AL-20MS | C_12_H_18_O_4_ | 225.1121 | 225.1122 | -0.44 | 4.79 | 93; 135; 181 | EB; FAE | 12-Hydroxyjasmonic acid | HMDB |
| AL-21MS | C_16_H_18_O_9_ | 353.0872 | 353.0867 | 1.41 | 4.41 | 135; 153; 179; 191 | EB; FAE; FHM | Chlorogenic acid | HMDB |
| AL-22MS | C_7_H_12_O_6_ | 191.0555 | 191.0553 | 1.04 | 4.43 | 85; 153; 191 | EB; FAE; FHM | Quinic acid | MoNA |
| AL-23MS | C_25_H_24_O_12_ | 515.1189 | 515.1176 | 2.52 | 4.9 | 179; 191; 351 | EB; FAE | 3,4-Dicaffeoylquinic acid | HMDB |
| AL-24MS | C_17_H_20_O_9_ | 367.1028 | 367.102 | 2.17 | 4.73 | 164; 173; 191; 193 | EB; FAE; FHM | 3-O-Feruloylquinic acid | Ncube et al., 2014^1^ |
| AL-25MS | C_15_H_18_O_9_ | 341.0872 | 341.0865 | 2.05 | 4.17 | 109; 135; 161; 179 | EB; FAE | Caffeic acid 3-glucoside | HMDB |
| AL-26MS | C_16_H_16_O_8_ | 335.0767 | 335.0761 | 1.79 | 4.58 | 93; 135; 161; 179 | EB; FAE; FHM | 5-O-Caffeoylshikimic acid | MoNA |
| AL-27MS | C_6_H_12_O_6_ | 179.0556 | 179.0553 | 1.67 | 1.04 | 85; 101; 113 | EB; FHM | BETA-D-glucose | HMDB |
| AL-28MS | C_12_H_22_O_11_ | 341.1075 | 341.1075 | 2.63 | 0.88 | 89; 119; 179; 191 | EB; FHM | D-Turanose | HMDB |
| AL-29MS | C_6_H_6_O_2_ | 109.0289 | 109.0289 | 0.00 | 4.12 | 109 | EB; FAE | Hidroquinone | HMDB |
| AL-30MS | C_9_H_6_O_4_ | 177.0188 | 177.0187 | 0.56 | 4.68 | 105; 135; 149; 177 | EB; FDC; FAE | Daphnetin | MoNA |
| AL-31MS | C_15_H_16_O_9_ | 339.0715 | 339.0708 | 2.06 | 4.32 | 135; 161; 177; 339 | EB; FAE; FHM | Esculin | MoNA |
| AL-32MS | C_15_H_22_O_9_ | 345.1185 | 345.1172 | 3.76 | 4.24 | 119; 165; 181 | EB; FHM | Aucubin | HMDB |
| AL-33MS | C_33_H_40_O_20_ | 755.2034 | 755.2004 | 3.97 | 4.48 | 163; 300; 325 | EB; FAE; FHM | Quercetin 3-O-rutinoside-4'-O-rhamnoside | HMDB |
| AL-34MS | C_27_H_30_O_16_ | 609.1455 | 609.1438 | 2.79 | 4.57 | 155; 255; 271; 300 | EB; FAE; FHM | Rutin | MoNA |
| AL-35MS | C_21_H_20_O_12_ | 463.0876 | 463.0866 | 2.15 | 4.76 | 161; 255; 271; 300 | EB; FAE; FHM | Quercetin-3-O-glucoside | MoNA |
| AL-36MS | C_27_H_30_O_15_ | 593.1506 | 593.1496 | 1.68 | 4.76 | 161; 227; 255; 284 | EB; FHM | Kaempferol-3-O-rutinoside | MoNA |
| AL-37MS | C_15_H_10_O_5_ | 283.0606 | 283.0596 | 3.53 | 6.40 | 117; 148; 240; 268 | EB; FDC | Apigenin | HMDB |
| AL-38MS | C_21_H_18_O_13_ | 477.0669 | 477.0654 | 3.14 | 4.88 | 151; 229; 271; 301 | EB; FAE; FHM | Quercetin-3-O-glucuronide | MoNA |

^1^Ncube, E. N.; Mhlongo, M. I.; Piater, L. A.; Steenkamp, P. A.; Dubery, I. A.; Madala, N. E. 2014. Analyses of chlorogenic acids and related cinnamic acid derivatives from Nicotiana tabacumtissues with the aid of UPLC-QTOF-MS/MS based on the in-source collision-induced dissociation method. Chemistry Central Journal, v. 8, p. 1-10.

**Figure 12S** - ^1^H-NMR spectrum (CDCl_3_. 300 MHz) of compound **5**.


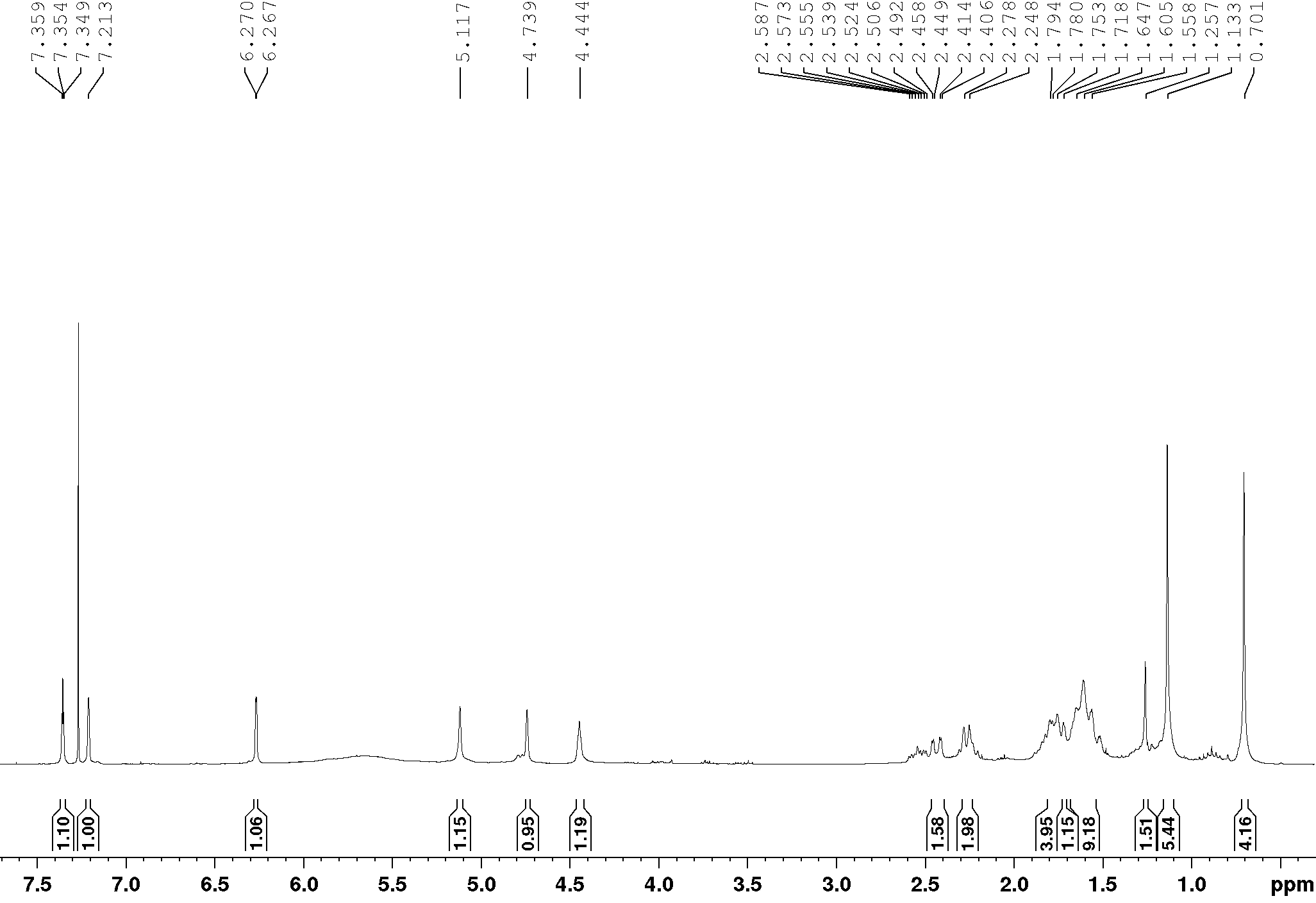


**Figure 13S** - ^13^C-NMR spectrum (CDCl_3_. 75 MHz) of compound **5**.


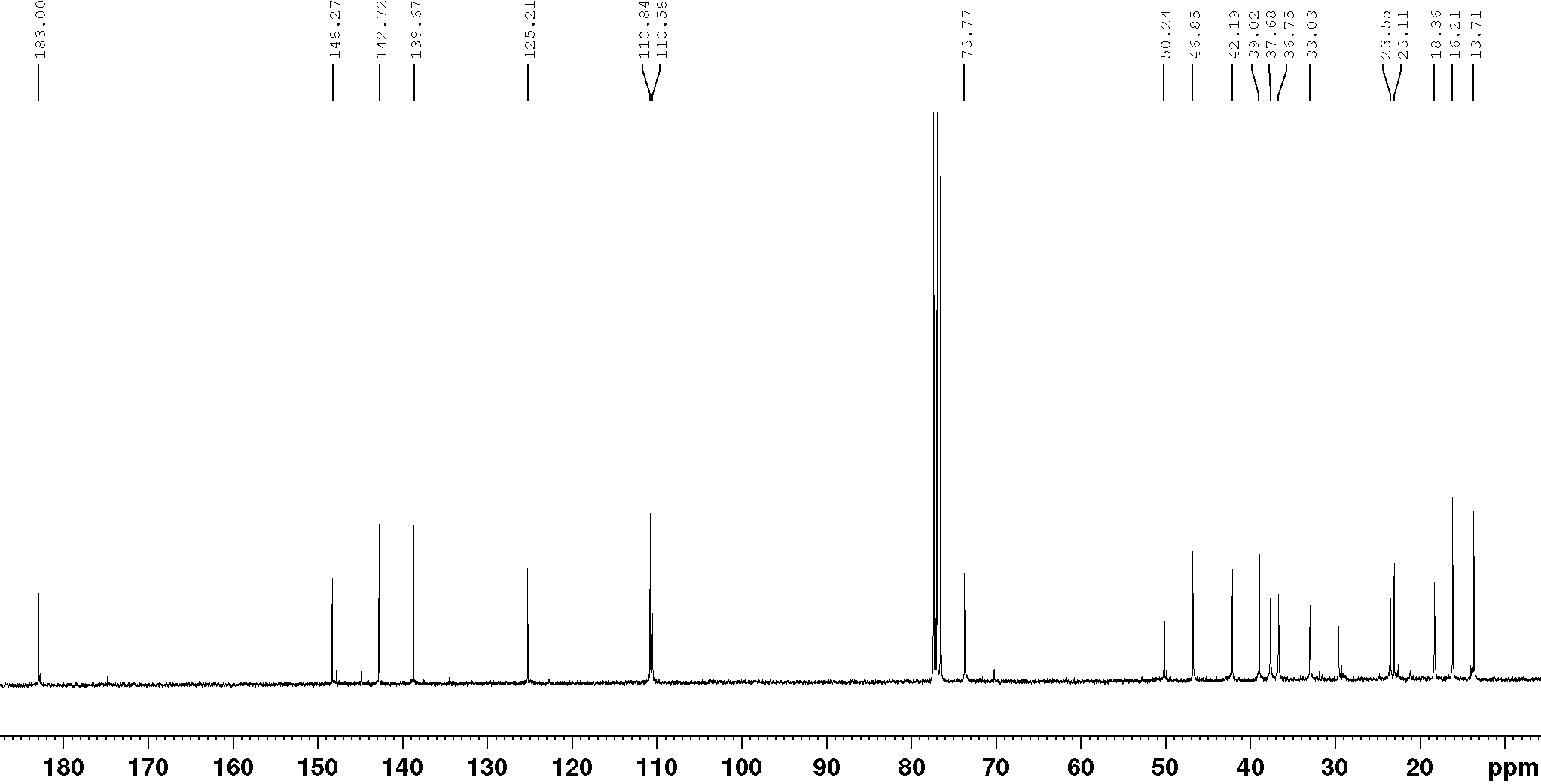


**Figure 14S -** HSQC spectrum (CDCl_3_. 300 and 75 MHz) of compound **5**.


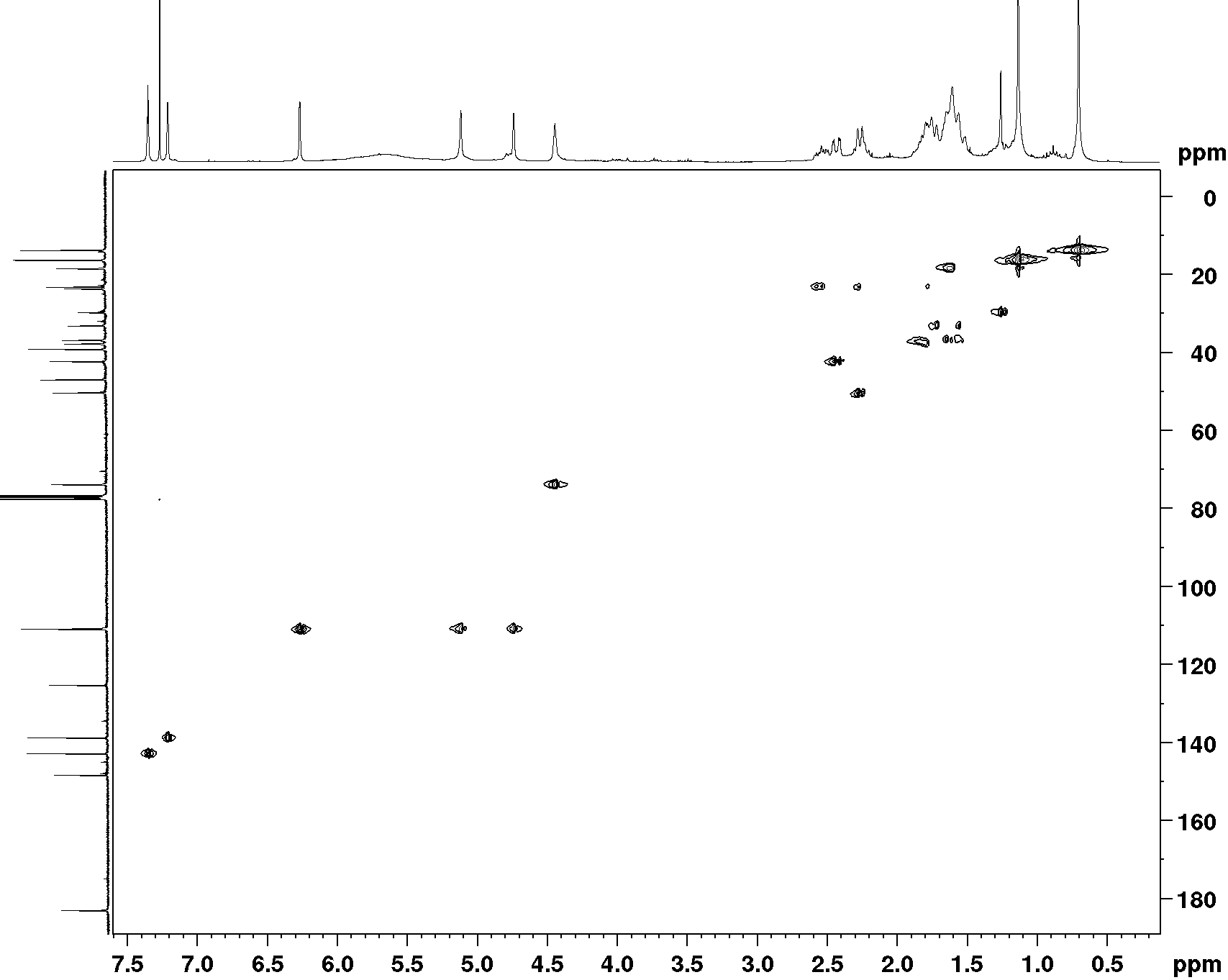


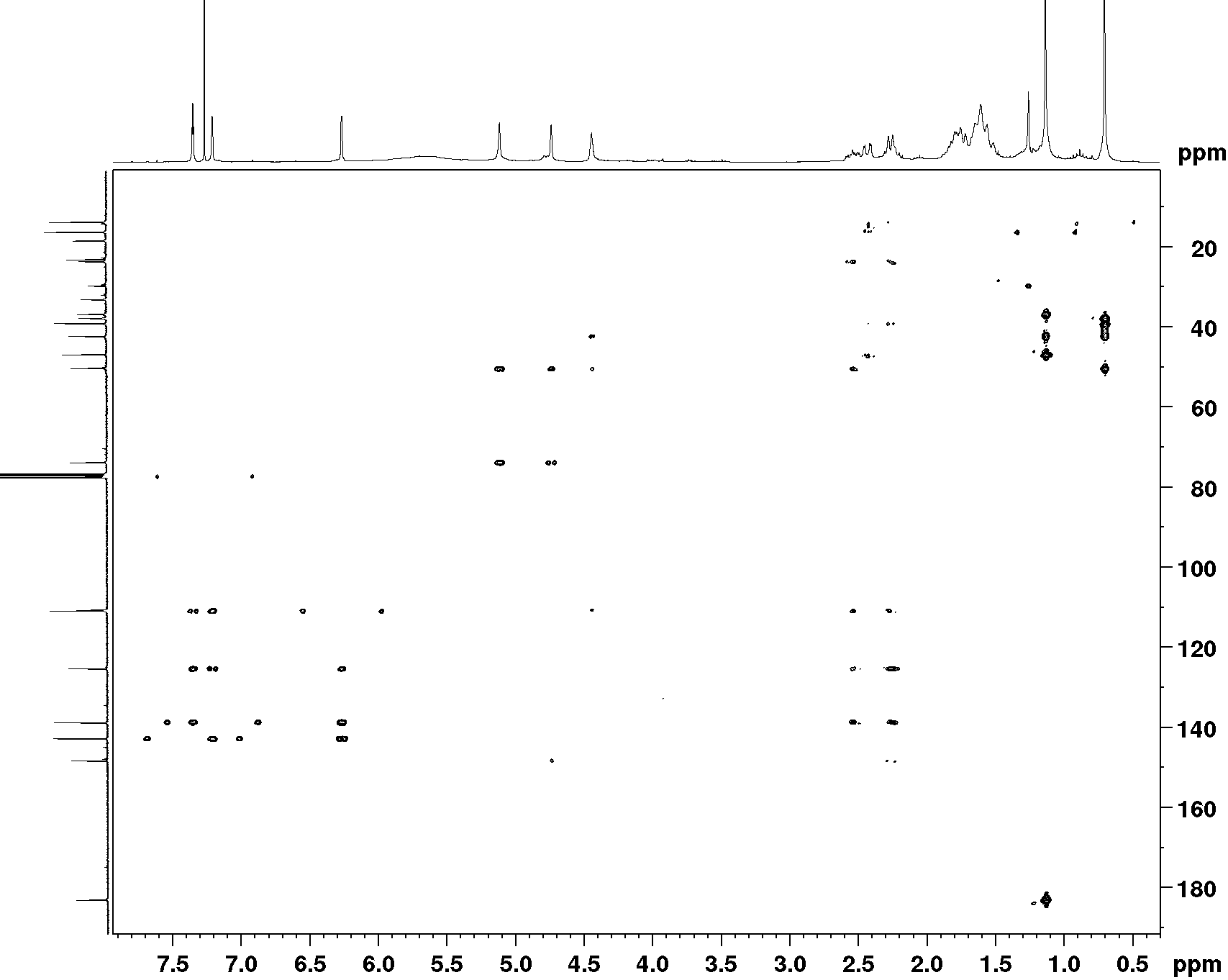


**Figure 15S** - HMBC spectrum (CDCl_3_, 300 and 75 MHz) of compound **5**.

**Figure 16S -** TOCSY spectrum and expansion (CDCl_3_. 300 MHz) of compound **5**.


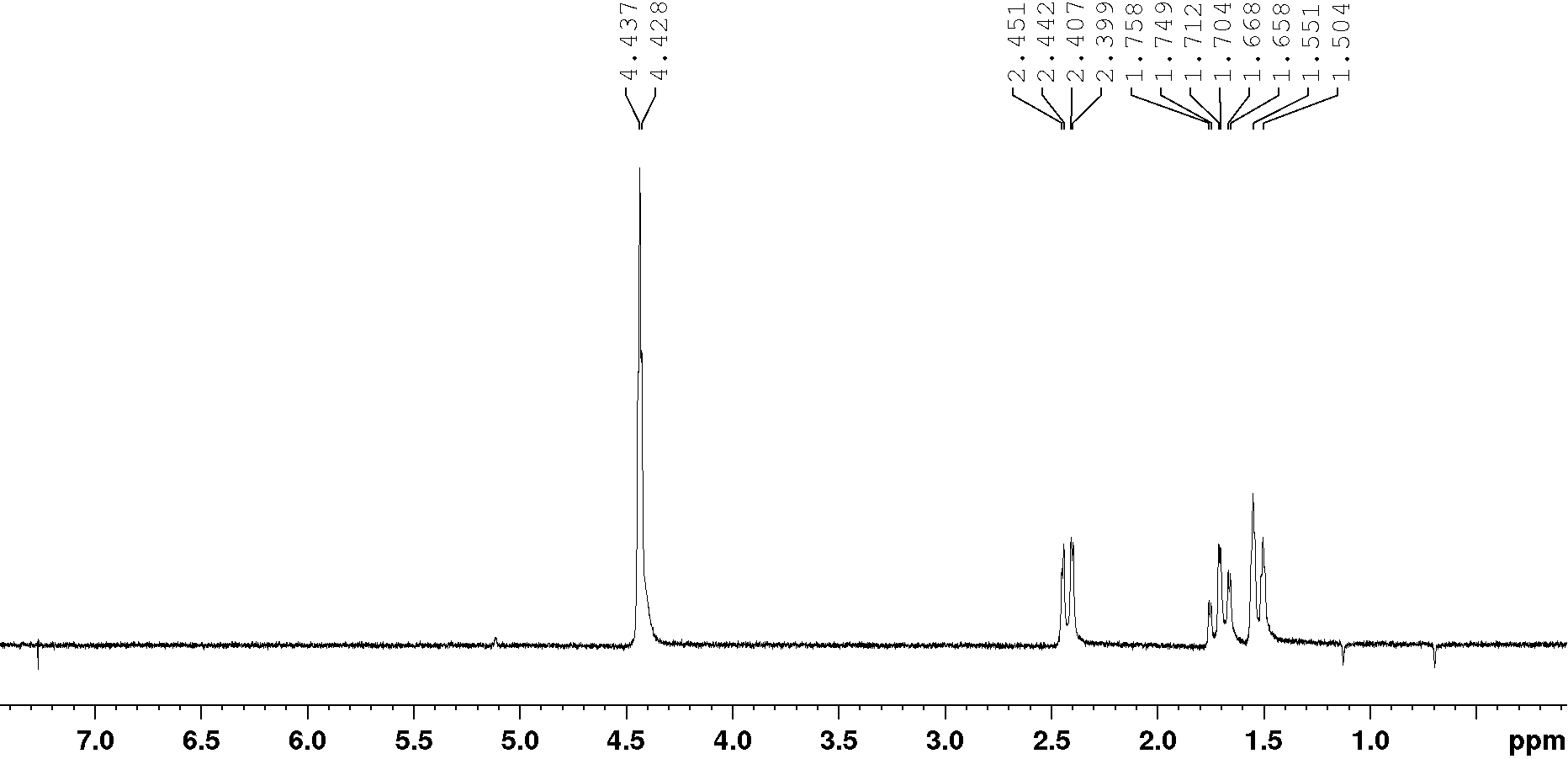


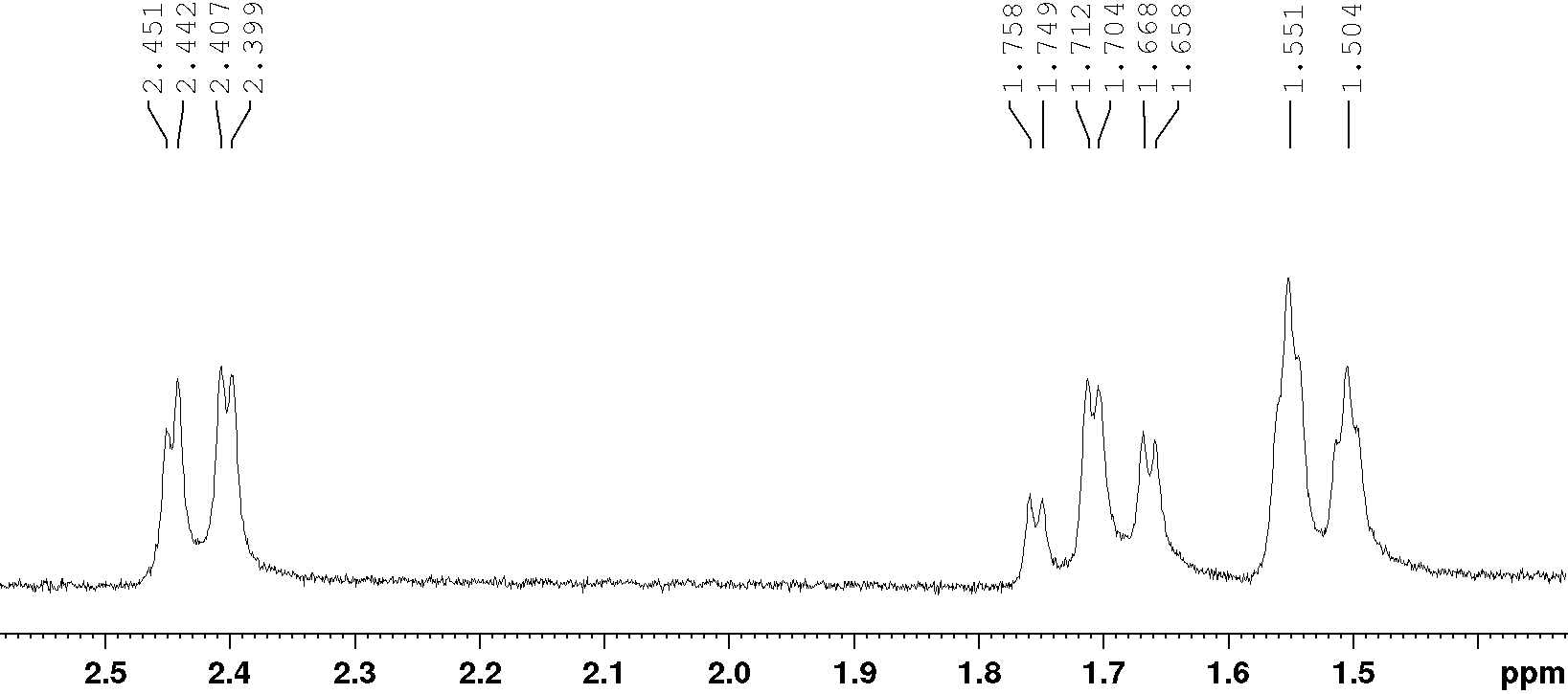


**Figure 17S** - pipHSQMBC spectrum for compound 5.


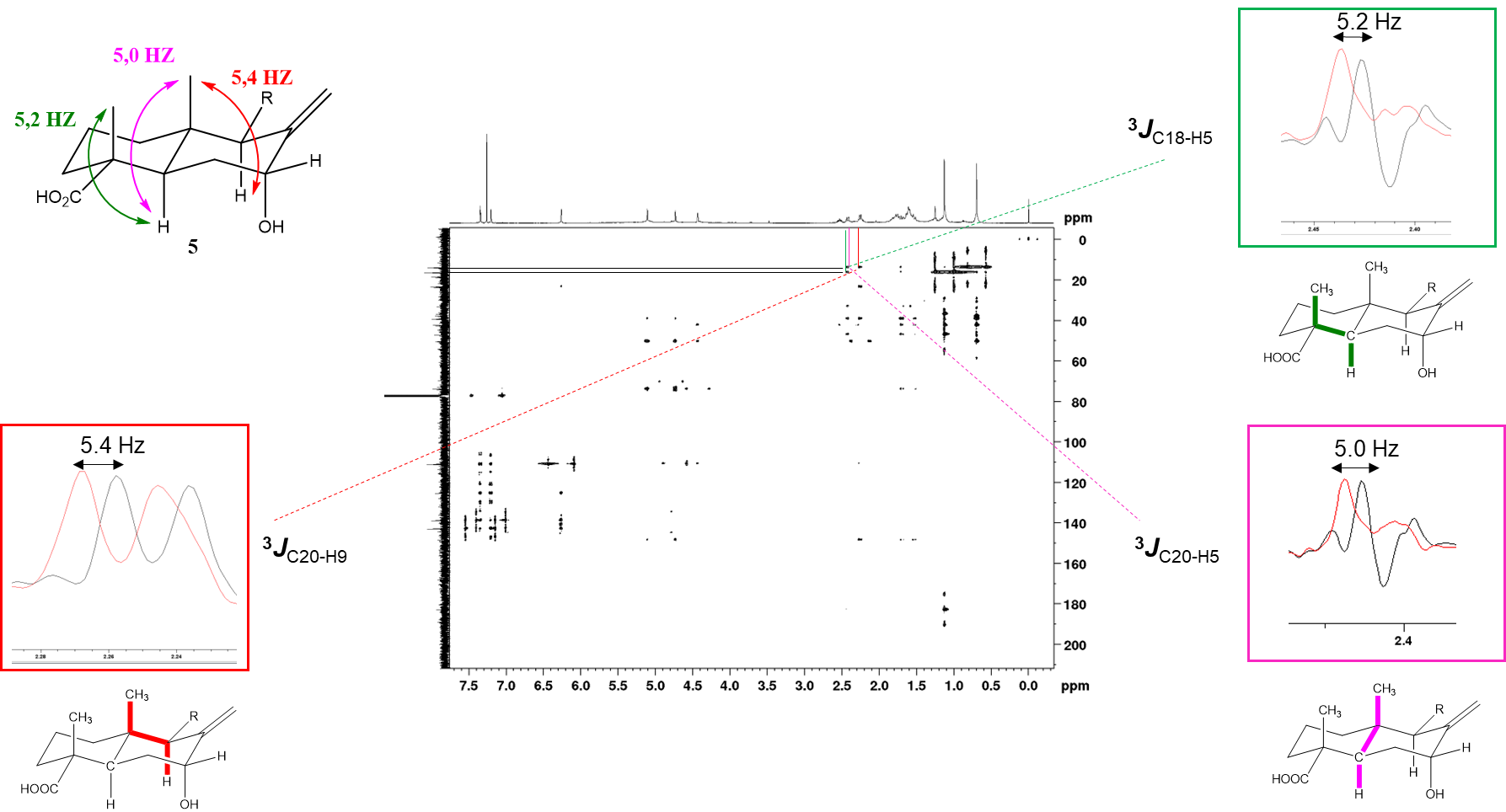


**Figure 18S** - ^1^H-NMR spectrum (CDCl_3_. 300 MHz) of compound 6.


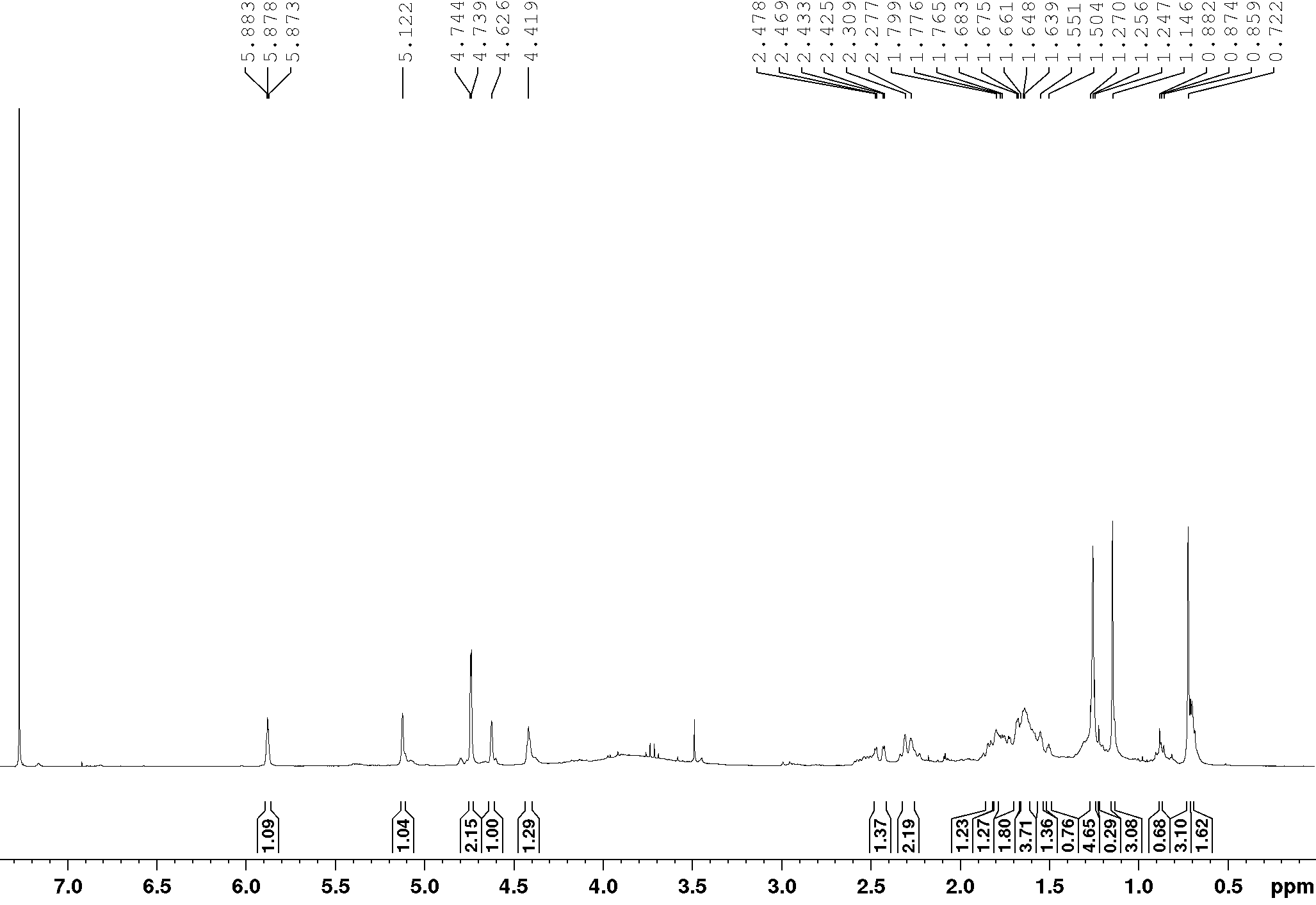


**Figure 19S** - ^13^C-NMR spectrum (CDCl_3_. 75 MHz) of compound **6**.


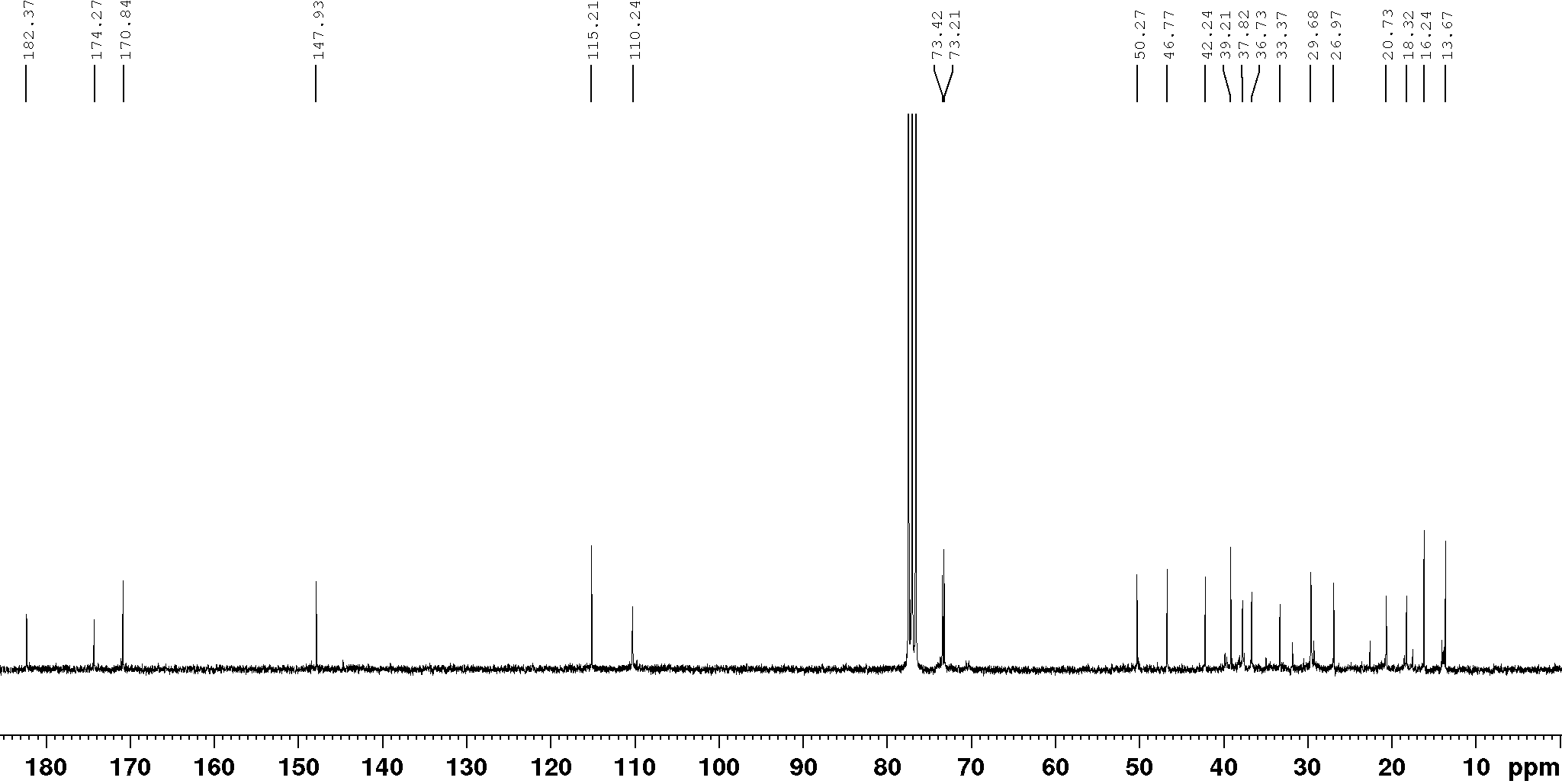


**Figure 20S -** HSQC spectrum (CDCl_3_, 300 and 75 MHz)) for **6**.


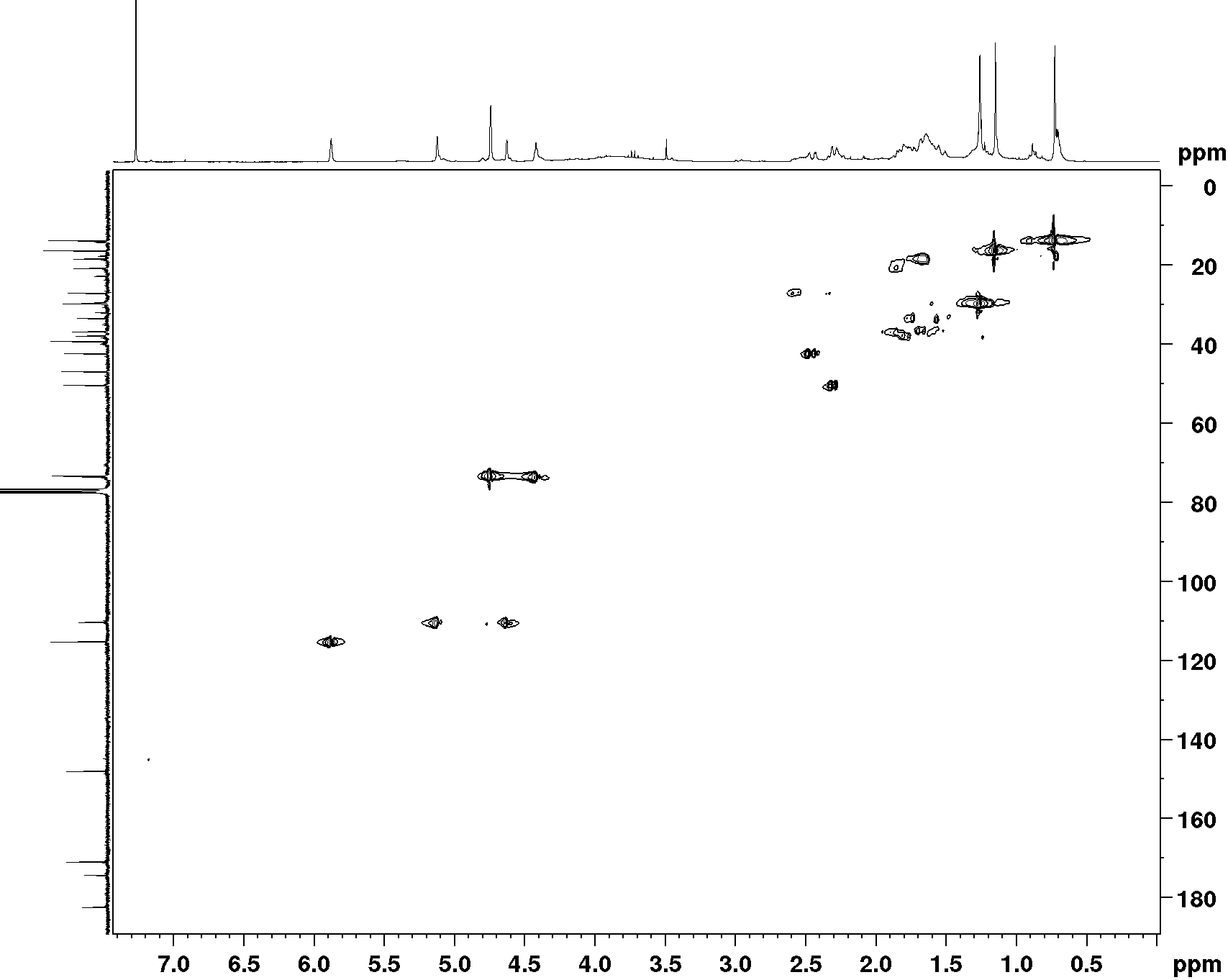


**Figure 21S -** HMBC spectrum (CDCl_3_. 300 and 75 MHz) for **6**.


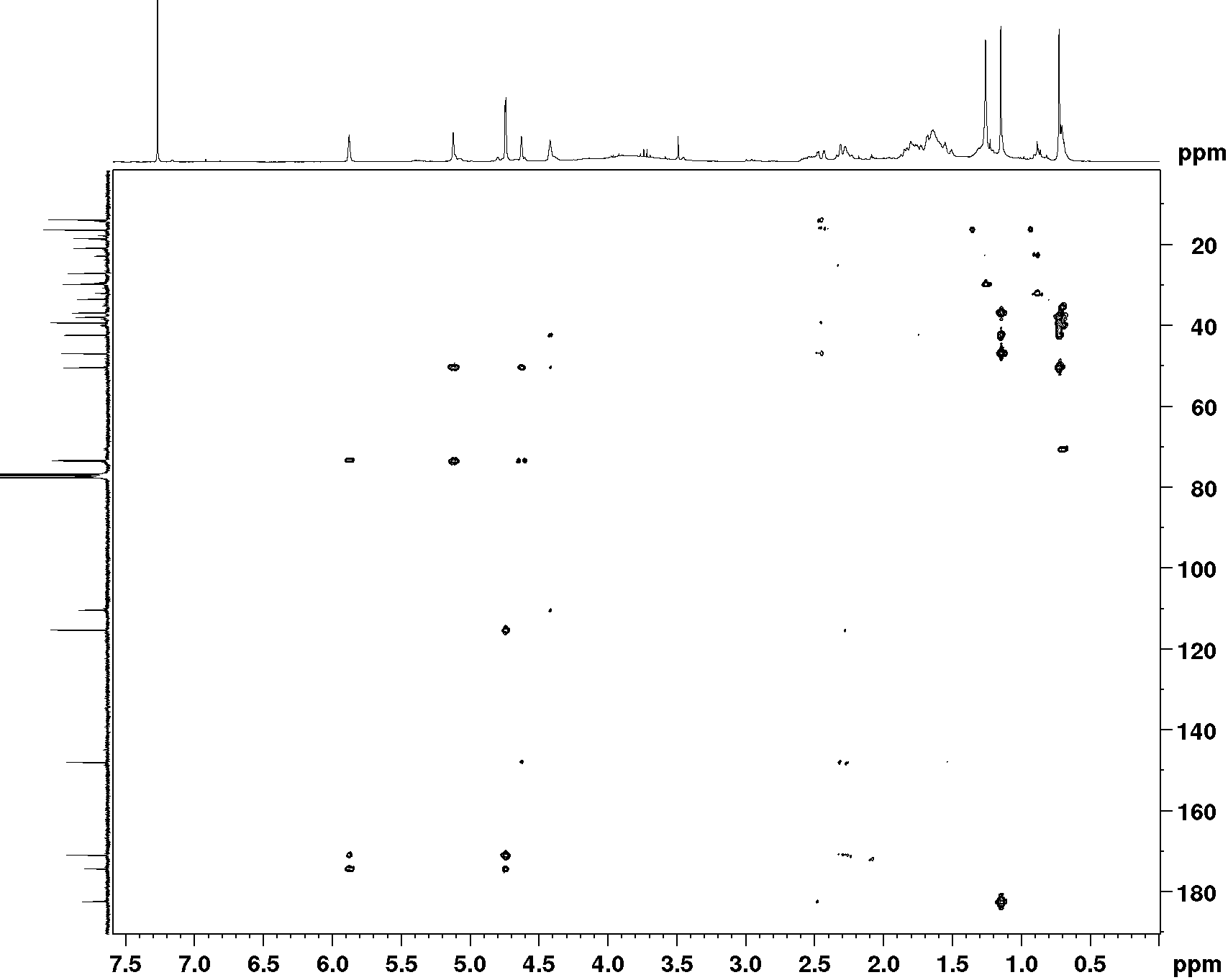


**Figure 22S** - pipHSQMBC spectrum for compound 6.


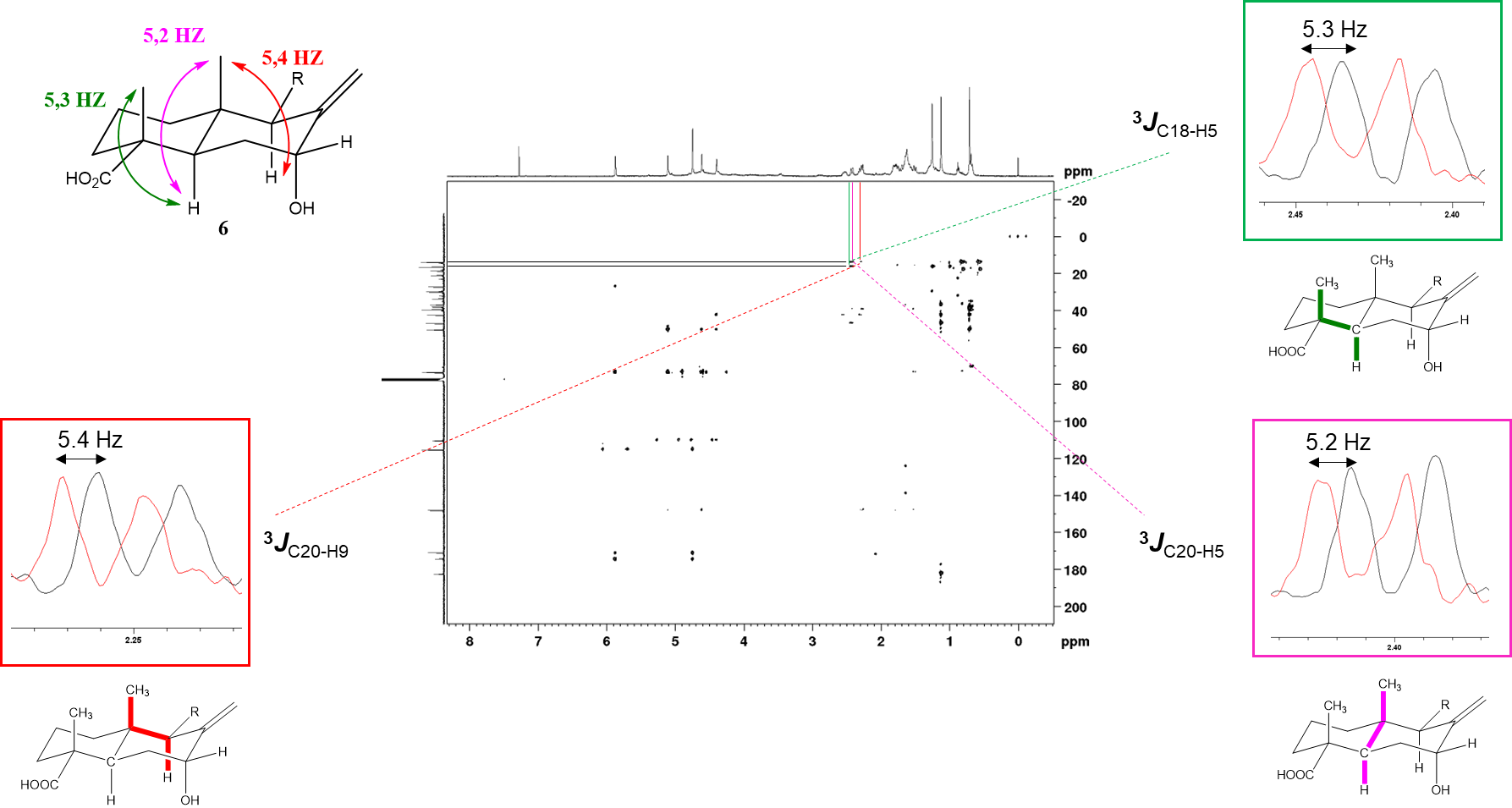


**Tabela 4S -** Inhibition of cell growth (%) of extracts. fractions and substances from *A. laetevirens* against the colon carcinoma line (HCT-116). at concentrations of 5 and 50 μg (mean ± DP).

| **Extract/Fractions** | **5μM** | **50μM** |
| --- | --- | --- |
| ***A. laetevirens*** |  |  |
| AL-CE | 8.4 ± 0.7 | 24.3 ± 7.9 |
| AL-HEX | 23.8 ± 4.0 | 36.7 ± 9.8 |
| AL-DC | 5.3 ± 3.3 | 40.6 ± 5.6 |
| AL-EAF | 3.6 ± 1.6 | 25.3 ± 4.8 |
| AL-HM | 11.8 ± 2.1 | 12.1 ± 7.3 |

**Tabela 5S -** Minimum Inhibitory Concentration (MIC) Values ​​of extracts. fractions and substances from *A. laetevirens* against *M. tuberculosis* (ATCC 27294).

| 1. ***laetevirens M. tuberculosis* MIC (μg mL^-1^)** | |
| --- | --- |
| AL-CE | > 250 |
| AL-HEX | > 250 |
| AL-DC | > 250 |
| AL-EAF | 250.0 |
| AL-HM | > 250 |
| 5  6 | > 250  > 250 |
| 7 | 125.0 |
| 8 | 125.0 |
| 9 | > 250 |
| **Standard (Isoniazid)** | 0.06 |

**Atomic coordinates**

(4*R*,5*R*,7*R*,9*R*,10*R*) – labdane (**5**)

B3PW91/PCM(CHCl_3_)/6-311G(d,p) level

Conf1 (-1269.090056 Hartree)

| Symbol | X | Y | Z |
| --- | --- | --- | --- |
| C | -0.59834 | 3.052347 | -0.36652 |
| C | -1.95568 | 2.407838 | -0.61525 |
| C | 0.528411 | 2.023432 | -0.42316 |
| C | 0.362692 | 0.875449 | 0.593129 |
| C | -1.06297 | 0.263832 | 0.402129 |
| C | -2.2626 | 1.270386 | 0.389701 |
| C | 1.367745 | -0.29047 | 0.247474 |
| C | 1.123855 | -1.45589 | 1.184923 |
| C | -0.2736 | -2.02602 | 1.110324 |
| C | 2.002136 | -1.9601 | 2.053013 |
| C | -2.64144 | 1.842449 | 1.759307 |
| C | -3.4735 | 0.505452 | -0.14599 |
| O | -4.51024 | 0.363658 | 0.495976 |
| O | -3.31548 | 0.015404 | -1.35668 |
| C | 0.667062 | 1.394167 | 2.006376 |
| C | 2.83859 | 0.128332 | 0.141597 |
| C | 3.69114 | -0.86857 | -0.6604 |
| C | 5.122188 | -0.4465 | -0.78353 |
| C | 6.209569 | -1.03576 | -0.2177 |
| C | 5.641168 | 0.681439 | -1.50945 |
| C | 6.984515 | 0.675927 | -1.3236 |
| O | 7.35096 | -0.36654 | -0.53607 |
| H | -0.5899 | 3.576341 | 0.595364 |
| H | -0.42448 | 3.821605 | -1.12657 |
| H | -1.96697 | 1.994151 | -1.62818 |
| H | -2.75402 | 3.157033 | -0.56593 |
| H | 1.482671 | 2.533108 | -0.26003 |
| H | 0.565739 | 1.596108 | -1.43498 |
| H | 1.062296 | -0.63751 | -0.75039 |
| O | -0.53339 | -2.59408 | -0.17684 |
| H | 3.010838 | -1.577 | 2.162635 |
| H | 1.725661 | -2.78713 | 2.701319 |
| H | -3.56066 | 2.426455 | 1.676373 |
| H | -2.82213 | 1.055684 | 2.49369 |
| H | -1.86293 | 2.497159 | 2.146971 |
| H | -4.13519 | -0.47849 | -1.64357 |
| H | 1.729403 | 1.643022 | 2.086815 |
| H | 0.113268 | 2.301663 | 2.24734 |
| H | 0.450119 | 0.655202 | 2.780284 |
| H | 2.90635 | 1.098629 | -0.35595 |
| H | 3.282017 | 0.267659 | 1.132785 |
| H | 3.645003 | -1.85957 | -0.20075 |
| H | 3.257047 | -0.97222 | -1.66264 |
| H | 6.332647 | -1.90524 | 0.408451 |
| H | 5.080632 | 1.393875 | -2.09683 |
| H | 7.785167 | 1.31021 | -1.66882 |
| H | -0.39243 | -2.79668 | 1.883951 |
| H | 0.154067 | -3.24548 | -0.34611 |
| C | -1.30834 | -0.9291 | 1.329149 |
| H | -1.04731 | -0.14379 | -0.61513 |
| H | -1.279 | -0.62887 | 2.380663 |
| H | -2.29932 | -1.36151 | 1.152879 |
| C | -6.48205 | -1.42556 | -1.4656 |
| O | -5.48315 | -1.30104 | -2.15397 |
| O | -6.65166 | -0.94381 | -0.26462 |
| H | -7.36139 | -1.97909 | -1.81695 |
| H | -5.82695 | -0.44259 | 0.025332 |

Conf2 (-1269.089580 Hartree)

| Symbol | X | Y | Z |
| --- | --- | --- | --- |
| C | -0.6054 | 2.980549 | -0.81087 |
| C | -1.97154 | 2.319151 | -0.93223 |
| C | 0.511799 | 1.943732 | -0.71658 |
| C | 0.350528 | 0.973843 | 0.469834 |
| C | -1.08247 | 0.355222 | 0.397051 |
| C | -2.27487 | 1.361022 | 0.243888 |
| C | 1.343064 | -0.24197 | 0.297479 |
| C | 1.102343 | -1.25029 | 1.402078 |
| C | -0.30171 | -1.80752 | 1.435528 |
| C | 1.990463 | -1.63404 | 2.320349 |
| C | -2.62681 | 2.149436 | 1.510555 |
| C | -3.46638 | 0.50606 | -0.19334 |
| O | -3.46801 | -0.09666 | -1.26357 |
| O | -4.47282 | 0.455972 | 0.650215 |
| C | 0.679306 | 1.702591 | 1.781011 |
| C | 2.815694 | 0.144691 | 0.115095 |
| C | 3.646545 | -0.96144 | -0.55583 |
| C | 5.085532 | -0.58753 | -0.73067 |
| C | 5.725166 | -0.29313 | -1.89432 |
| C | 6.083109 | -0.43806 | 0.294448 |
| C | 7.229306 | -0.07239 | -0.33164 |
| O | 7.030162 | 0.021249 | -1.6705 |
| H | -0.57697 | 3.653474 | 0.053282 |
| H | -0.43646 | 3.61471 | -1.68765 |
| H | -2.00544 | 1.741296 | -1.8609 |
| H | -2.76231 | 3.075547 | -0.99569 |
| H | 1.472634 | 2.462291 | -0.64906 |
| H | 0.531022 | 1.36097 | -1.64783 |
| H | 1.023759 | -0.73298 | -0.63346 |
| O | -0.58827 | -2.56169 | 0.253663 |
| H | 3.005668 | -1.25447 | 2.353776 |
| H | 1.716669 | -2.35343 | 3.087159 |
| H | -3.54975 | 2.713674 | 1.358253 |
| H | -2.78034 | 1.498635 | 2.372709 |
| H | -1.84289 | 2.863052 | 1.760175 |
| H | -5.19652 | -0.13311 | 0.289183 |
| H | 0.486806 | 1.087041 | 2.662043 |
| H | 1.740516 | 1.96786 | 1.798624 |
| H | 0.120424 | 2.631359 | 1.893721 |
| H | 2.890232 | 1.034068 | -0.51483 |
| H | 3.2715 | 0.417328 | 1.072682 |
| H | 3.576428 | -1.88471 | 0.028789 |
| H | 3.212752 | -1.18333 | -1.53695 |
| H | 5.40474 | -0.26897 | -2.92392 |
| H | 5.956591 | -0.59023 | 1.35631 |
| H | 8.22733 | 0.146092 | 0.012981 |
| H | -0.41534 | -2.45032 | 2.318846 |
| H | 0.090222 | -3.23933 | 0.175873 |
| C | -1.32236 | -0.67806 | 1.500184 |
| H | -1.09152 | -0.20255 | -0.54725 |
| H | -1.2717 | -0.22095 | 2.492648 |
| H | -2.31894 | -1.12268 | 1.408595 |
| C | -6.34532 | -1.71333 | -1.32355 |
| O | -6.41019 | -1.11586 | -0.26174 |
| O | -5.3524 | -1.67179 | -2.16821 |
| H | -7.15682 | -2.3649 | -1.66993 |
| H | -4.62613 | -1.06645 | -1.81792 |

Conf3 (-1269.089362 Hartree)

| Symbol | X | Y | Z |
| --- | --- | --- | --- |
| C | -0.58086 | 3.030395 | -0.52865 |
| C | -1.94017 | 2.379212 | -0.74803 |
| C | 0.54116 | 1.994414 | -0.52459 |
| C | 0.366426 | 0.902688 | 0.549353 |
| C | -1.06166 | 0.288912 | 0.386961 |
| C | -2.25636 | 1.29931 | 0.315588 |
| C | 1.366027 | -0.28503 | 0.265088 |
| C | 1.114882 | -1.40248 | 1.257205 |
| C | -0.28738 | -1.96483 | 1.214647 |
| C | 1.993111 | -1.87575 | 2.142637 |
| C | -2.63726 | 1.946915 | 1.650579 |
| C | -3.46907 | 0.510491 | -0.18045 |
| O | -4.5045 | 0.402249 | 0.469698 |
| O | -3.31023 | -0.04212 | -1.36371 |
| C | 0.671534 | 1.490841 | 1.934678 |
| C | 2.838609 | 0.123371 | 0.140871 |
| C | 3.684005 | -0.90303 | -0.63088 |
| C | 5.120338 | -0.49975 | -0.75558 |
| C | 5.765959 | -0.08735 | -1.87937 |
| C | 6.107578 | -0.43978 | 0.288305 |
| C | 7.25461 | -0.00299 | -0.28879 |
| O | 7.065315 | 0.217876 | -1.61415 |
| H | -0.57373 | 3.607178 | 0.402811 |
| H | -0.39986 | 3.755815 | -1.32904 |
| H | -1.94876 | 1.910822 | -1.73684 |
| H | -2.73525 | 3.133372 | -0.74303 |
| H | 1.497 | 2.507789 | -0.38524 |
| H | 0.580536 | 1.513964 | -1.51213 |
| H | 1.060003 | -0.67838 | -0.71535 |
| O | -0.55267 | -2.59871 | -0.04043 |
| H | 3.006373 | -1.49901 | 2.228543 |
| H | 1.712795 | -2.67013 | 2.828927 |
| H | -3.55518 | 2.527139 | 1.533578 |
| H | -2.82157 | 1.203024 | 2.427378 |
| H | -1.85842 | 2.62082 | 2.003019 |
| H | -4.12346 | -0.55902 | -1.62483 |
| H | 1.733176 | 1.746782 | 2.0004 |
| H | 0.114773 | 2.407006 | 2.131889 |
| H | 0.459155 | 0.789633 | 2.744195 |
| H | 2.914415 | 1.072831 | -0.39393 |
| H | 3.281452 | 0.298302 | 1.126823 |
| H | 3.619135 | -1.88117 | -0.14306 |
| H | 3.259635 | -1.02894 | -1.63293 |
| H | 5.453359 | 0.033372 | -2.90457 |
| H | 5.973551 | -0.69574 | 1.329045 |
| H | 8.247176 | 0.191796 | 0.084554 |
| H | -0.41027 | -2.69273 | 2.027995 |
| H | 0.13001 | -3.26312 | -0.17506 |
| C | -1.3153 | -0.8512 | 1.375429 |
| H | -1.04427 | -0.17359 | -0.60656 |
| H | -1.28463 | -0.49656 | 2.409744 |
| H | -2.3085 | -1.28698 | 1.221756 |
| C | -6.48354 | -1.49094 | -1.37541 |
| O | -5.48293 | -1.41234 | -2.06762 |
| O | -6.65755 | -0.92884 | -0.21043 |
| H | -7.36361 | -2.06332 | -1.69341 |
| H | -5.83047 | -0.41644 | 0.050885 |

Conf4 (-1269.089315 Hartree)

| Symbol | X | Y | Z |
| --- | --- | --- | --- |
| C | -0.61117 | 3.031059 | -0.42507 |
| C | -1.96947 | 2.377606 | -0.64622 |
| C | 0.519851 | 2.006217 | -0.47338 |
| C | 0.370732 | 0.875689 | 0.565154 |
| C | -1.05503 | 0.258907 | 0.39757 |
| C | -2.25883 | 1.257024 | 0.384429 |
| C | 1.377567 | -0.29175 | 0.226787 |
| C | 1.134477 | -1.45794 | 1.163888 |
| C | -0.25651 | -2.03512 | 1.076886 |
| C | 2.000192 | -1.93779 | 2.056275 |
| C | -2.62372 | 1.853085 | 1.74743 |
| C | -3.47708 | 0.488419 | -0.12877 |
| O | -4.53874 | 0.433361 | 0.48426 |
| O | -3.30029 | -0.10095 | -1.29328 |
| C | 0.686683 | 1.418809 | 1.966258 |
| C | 2.847799 | 0.131157 | 0.128971 |
| C | 3.709544 | -0.87072 | -0.65664 |
| C | 5.141603 | -0.44835 | -0.76596 |
| C | 6.222104 | -1.03095 | -0.18041 |
| C | 5.669386 | 0.672463 | -1.49667 |
| C | 7.010278 | 0.669998 | -1.29361 |
| O | 7.3673 | -0.36403 | -0.49079 |
| H | -0.5948 | 3.575014 | 0.525597 |
| H | -0.44922 | 3.785458 | -1.20247 |
| H | -1.99073 | 1.944186 | -1.65118 |
| H | -2.76999 | 3.124494 | -0.60107 |
| H | 1.473219 | 2.523229 | -0.33015 |
| H | 0.548783 | 1.559844 | -1.47715 |
| H | 1.078986 | -0.64322 | -0.77098 |
| O | -0.41103 | -2.59694 | -0.22865 |
| H | 2.99678 | -1.53045 | 2.186631 |
| H | 1.729039 | -2.77018 | 2.699221 |
| H | -3.55357 | 2.419846 | 1.668438 |
| H | -2.77711 | 1.079611 | 2.502419 |
| H | -1.84934 | 2.528287 | 2.106692 |
| H | -4.13344 | -0.5772 | -1.57252 |
| H | 1.744361 | 1.691753 | 2.025477 |
| H | 0.117088 | 2.316529 | 2.206454 |
| H | 0.49992 | 0.683919 | 2.751962 |
| H | 2.91637 | 1.098006 | -0.37569 |
| H | 3.283122 | 0.280014 | 1.122313 |
| H | 3.657167 | -1.85808 | -0.19042 |
| H | 3.286725 | -0.9828 | -1.66256 |
| H | 6.337592 | -1.89439 | 0.45545 |
| H | 5.116014 | 1.378141 | -2.09883 |
| H | 7.814909 | 1.30123 | -1.6352 |
| H | -0.37097 | -2.82252 | 1.83365 |
| H | -1.33987 | -2.8207 | -0.3424 |
| C | -1.2818 | -0.92832 | 1.335206 |
| H | -1.04533 | -0.15828 | -0.6158 |
| H | -1.21538 | -0.62867 | 2.385886 |
| H | -2.28997 | -1.34352 | 1.202681 |
| C | -6.52316 | -1.41022 | -1.41249 |
| O | -5.48841 | -1.39765 | -2.05822 |
| O | -6.7194 | -0.81762 | -0.26662 |
| H | -7.41464 | -1.9468 | -1.75901 |
| H | -5.88187 | -0.33781 | 0.021041 |

Conf5 (-1269.089056 Hartree)

| Symbol | X | Y | Z |
| --- | --- | --- | --- |
| C | -0.68429 | 2.957901 | -0.56087 |
| C | -2.03372 | 2.265228 | -0.69652 |
| C | 0.463631 | 1.951771 | -0.56958 |
| C | 0.369256 | 0.90698 | 0.559834 |
| C | -1.05233 | 0.259389 | 0.50054 |
| C | -2.27439 | 1.213427 | 0.421547 |
| C | 1.379457 | -0.27288 | 0.276874 |
| C | 1.184678 | -1.37595 | 1.298709 |
| C | -0.20942 | -1.95634 | 1.324905 |
| C | 2.098159 | -1.80386 | 2.170022 |
| C | -2.64672 | 1.899912 | 1.744075 |
| C | -3.47319 | 0.409585 | -0.08725 |
| O | -3.36626 | -0.66645 | -0.67356 |
| O | -4.63208 | 1.000433 | 0.0956 |
| C | 0.73081 | 1.566383 | 1.898418 |
| C | 2.840298 | 0.159448 | 0.102099 |
| C | 3.682533 | -0.87031 | -0.66844 |
| C | 5.109335 | -0.44961 | -0.83648 |
| C | 6.209285 | -1.01514 | -0.27085 |
| C | 5.611186 | 0.651367 | -1.61423 |
| C | 6.957978 | 0.655603 | -1.45474 |
| O | 7.342643 | -0.35594 | -0.63616 |
| H | -0.65628 | 3.566265 | 0.34981 |
| H | -0.56064 | 3.659456 | -1.39278 |
| H | -2.06579 | 1.757372 | -1.66912 |
| H | -2.85208 | 2.99139 | -0.68835 |
| H | 1.411455 | 2.493567 | -0.50187 |
| H | 0.466675 | 1.42838 | -1.53583 |
| H | 1.053904 | -0.69885 | -0.68298 |
| O | -0.45186 | -2.61476 | 0.07753 |
| H | 3.10066 | -1.39361 | 2.222174 |
| H | 1.861537 | -2.5967 | 2.873911 |
| H | -3.55438 | 2.491865 | 1.622015 |
| H | -2.82624 | 1.172582 | 2.53971 |
| H | -1.8536 | 2.567218 | 2.077716 |
| H | -5.36989 | 0.458374 | -0.30792 |
| H | 0.132794 | 2.454858 | 2.102994 |
| H | 0.618828 | 0.884993 | 2.744236 |
| H | 1.77658 | 1.886984 | 1.880329 |
| H | 2.880105 | 1.10225 | -0.44908 |
| H | 3.311408 | 0.359877 | 1.069945 |
| H | 3.64716 | -1.83888 | -0.16255 |
| H | 3.230153 | -1.02307 | -1.65605 |
| H | 6.346583 | -1.86096 | 0.384083 |
| H | 5.037473 | 1.340085 | -2.21699 |
| H | 7.749996 | 1.27812 | -1.83914 |
| H | -0.28137 | -2.68983 | 2.138594 |
| H | -1.38369 | -2.50343 | -0.1378 |
| C | -1.21723 | -0.83239 | 1.559448 |
| H | -1.06505 | -0.25449 | -0.46742 |
| H | -1.07707 | -0.43727 | 2.570044 |
| H | -2.22776 | -1.25583 | 1.528719 |
| C | -6.46565 | -1.46783 | -1.52911 |
| O | -6.62331 | -0.40668 | -0.94908 |
| O | -5.32461 | -2.06907 | -1.7302 |
| H | -7.3089 | -2.02945 | -1.94852 |
| H | -4.57677 | -1.52771 | -1.33128 |

Conf6 (-1269.088952 Hartree)

| Symbol | X | Y | Z |
| --- | --- | --- | --- |
| C | 0.319765 | -1.62645 | -2.75505 |
| C | 1.580161 | -0.88719 | -2.32661 |
| C | -0.87242 | -1.25027 | -1.87845 |
| C | -0.64476 | -1.53583 | -0.38042 |
| C | 0.694429 | -0.85073 | 0.045318 |
| C | 1.948544 | -1.14676 | -0.84691 |
| C | -1.76978 | -0.82937 | 0.471851 |
| C | -1.47606 | -1.01601 | 1.946974 |
| C | -0.14503 | -0.45522 | 2.390504 |
| C | -2.25608 | -1.6412 | 2.830854 |
| C | 2.555342 | -2.54386 | -0.67417 |
| C | 2.973952 | -0.07711 | -0.46479 |
| O | 2.768144 | 1.115887 | -0.67147 |
| O | 4.075152 | -0.5193 | 0.100441 |
| C | -0.70655 | -3.05011 | -0.13075 |
| C | -3.206 | -1.1921 | 0.078811 |
| C | -4.25727 | -0.17323 | 0.558732 |
| C | -4.16585 | 1.158314 | -0.12083 |
| C | -3.8408 | 2.354337 | 0.439504 |
| C | -4.4074 | 1.447877 | -1.50874 |
| C | -4.20991 | 2.7804 | -1.66586 |
| O | -3.8634 | 3.352044 | -0.48509 |
| H | 0.481927 | -2.71003 | -2.73651 |
| H | 0.095811 | -1.37606 | -3.79754 |
| H | 1.421522 | 0.187808 | -2.45528 |
| H | 2.427162 | -1.1656 | -2.96435 |
| H | -1.75751 | -1.78776 | -2.23214 |
| H | -1.08114 | -0.17933 | -2.00541 |
| H | -1.64451 | 0.244025 | 0.270264 |
| O | -0.10256 | 0.966825 | 2.240951 |
| H | -3.21241 | -2.08057 | 2.569854 |
| H | -1.94614 | -1.73938 | 3.867705 |
| H | 3.493071 | -2.6169 | -1.22997 |
| H | 2.775499 | -2.77203 | 0.369689 |
| H | 1.886039 | -3.31402 | -1.05473 |
| H | 4.679194 | 0.246084 | 0.325274 |
| H | -0.04917 | -3.6097 | -0.79606 |
| H | -0.44821 | -3.31608 | 0.89633 |
| H | -1.72268 | -3.41205 | -0.31336 |
| H | -3.28204 | -1.2622 | -1.00897 |
| H | -3.47353 | -2.18142 | 0.463231 |
| H | -5.25257 | -0.59607 | 0.378263 |
| H | -4.17429 | -0.03063 | 1.639735 |
| H | -3.58308 | 2.649479 | 1.444248 |
| H | -4.6954 | 0.749948 | -2.28107 |
| H | -4.27567 | 3.446979 | -2.51077 |
| H | 0.026282 | -0.72004 | 3.44279 |
| H | -0.85188 | 1.328208 | 2.723591 |
| C | 0.981519 | -1.02498 | 1.538756 |
| H | 0.514507 | 0.22042 | -0.10713 |
| H | 1.113144 | -2.07953 | 1.797813 |
| H | 1.907633 | -0.51285 | 1.820522 |
| C | 5.443532 | 2.653732 | 0.498404 |
| O | 5.705876 | 1.482703 | 0.718702 |
| O | 4.356073 | 3.104828 | -0.06246 |
| H | 6.135979 | 3.460761 | 0.767342 |
| H | 3.742275 | 2.339125 | -0.29474 |

Conf7 (-1269.088792 Hartree)

| Symbol | X | Y | Z |
| --- | --- | --- | --- |
| C | -0.60071 | 3.034579 | -0.56465 |
| C | -1.96527 | 2.384126 | -0.74572 |
| C | 0.516119 | 1.993377 | -0.54851 |
| C | 0.350759 | 0.931447 | 0.555869 |
| C | -1.08262 | 0.322001 | 0.431362 |
| C | -2.27496 | 1.336525 | 0.349472 |
| C | 1.34105 | -0.2681 | 0.287862 |
| C | 1.09876 | -1.3599 | 1.310077 |
| C | -0.3068 | -1.91495 | 1.302155 |
| C | 1.987824 | -1.81617 | 2.193644 |
| C | -2.63624 | 2.022884 | 1.671571 |
| C | -3.46279 | 0.517339 | -0.16029 |
| O | -3.45715 | -0.00141 | -1.27357 |
| O | -4.47434 | 0.402412 | 0.670715 |
| C | 0.677237 | 1.553592 | 1.921332 |
| C | 2.814215 | 0.129721 | 0.13695 |
| C | 3.648085 | -0.92139 | -0.6138 |
| C | 5.079504 | -0.51699 | -0.78352 |
| C | 6.171991 | -1.07626 | -0.19754 |
| C | 5.593522 | 0.561063 | -1.58494 |
| C | 6.93929 | 0.559705 | -1.41746 |
| O | 7.311874 | -0.43423 | -0.57215 |
| H | -0.57655 | 3.636621 | 0.350639 |
| H | -0.42814 | 3.737068 | -1.38704 |
| H | -1.99257 | 1.880811 | -1.71705 |
| H | -2.75664 | 3.142385 | -0.75395 |
| H | 1.476167 | 2.505391 | -0.43514 |
| H | 0.539656 | 1.486866 | -1.52336 |
| H | 1.021162 | -0.68328 | -0.67907 |
| O | -0.59551 | -2.57699 | 0.06681 |
| H | 3.003873 | -1.44205 | 2.255308 |
| H | 1.713578 | -2.59207 | 2.90316 |
| H | -3.55698 | 2.599437 | 1.556525 |
| H | -2.79823 | 1.306727 | 2.478482 |
| H | -1.85321 | 2.712381 | 1.983592 |
| H | -5.19493 | -0.15856 | 0.261759 |
| H | 0.47844 | 0.872277 | 2.750984 |
| H | 1.739519 | 1.811477 | 1.964565 |
| H | 0.122208 | 2.473753 | 2.104484 |
| H | 2.884461 | 1.066935 | -0.41987 |
| H | 3.272417 | 0.325798 | 1.111819 |
| H | 3.602569 | -1.88198 | -0.09328 |
| H | 3.19825 | -1.08354 | -1.6012 |
| H | 6.299744 | -1.90642 | 0.478998 |
| H | 5.028223 | 1.238879 | -2.20762 |
| H | 7.737905 | 1.167163 | -1.81211 |
| H | -0.42119 | -2.62254 | 2.134424 |
| H | 0.081951 | -3.24763 | -0.06312 |
| C | -1.32632 | -0.79198 | 1.451781 |
| H | -1.08821 | -0.16336 | -0.55218 |
| H | -1.2774 | -0.41222 | 2.476374 |
| H | -2.32326 | -1.22698 | 1.324548 |
| C | -6.32884 | -1.61688 | -1.47144 |
| O | -6.40362 | -1.09657 | -0.37047 |
| O | -5.33019 | -1.512 | -2.30387 |
| H | -7.13519 | -2.24514 | -1.86909 |
| H | -4.60895 | -0.93029 | -1.90664 |

Conf8 (-1269.088681 Hartree)

| Symbol | X | Y | Z |
| --- | --- | --- | --- |
| C | -0.66457 | 2.915598 | -0.73122 |
| C | -2.01929 | 2.22599 | -0.82525 |
| C | 0.476645 | 1.902546 | -0.67892 |
| C | 0.375055 | 0.92877 | 0.510871 |
| C | -1.05019 | 0.288963 | 0.490087 |
| C | -2.26591 | 1.244497 | 0.353822 |
| C | 1.378228 | -0.27284 | 0.298279 |
| C | 1.172883 | -1.31911 | 1.375755 |
| C | -0.22759 | -1.88218 | 1.437014 |
| C | 2.084716 | -1.71399 | 2.264247 |
| C | -2.6333 | 2.012191 | 1.632434 |
| C | -3.46889 | 0.417352 | -0.10584 |
| O | -3.36787 | -0.69692 | -0.61723 |
| O | -4.62385 | 1.028075 | 0.029531 |
| C | 0.741116 | 1.663423 | 1.808426 |
| C | 2.841543 | 0.143558 | 0.105322 |
| C | 3.679634 | -0.92008 | -0.62259 |
| C | 5.107926 | -0.51055 | -0.80567 |
| C | 5.720964 | -0.1524 | -1.96576 |
| C | 6.12053 | -0.38516 | 0.207977 |
| C | 7.248055 | 0.030814 | -0.42056 |
| O | 7.023499 | 0.178938 | -1.7505 |
| H | -0.63255 | 3.576226 | 0.14206 |
| H | -0.53576 | 3.565493 | -1.6032 |
| H | -2.05643 | 1.661883 | -1.76614 |
| H | -2.83235 | 2.957314 | -0.85949 |
| H | 1.428162 | 2.440811 | -0.64466 |
| H | 0.475445 | 1.321886 | -1.61177 |
| H | 1.053688 | -0.74726 | -0.63911 |
| O | -0.48447 | -2.60547 | 0.228864 |
| H | 3.092231 | -1.31422 | 2.291701 |
| H | 1.842188 | -2.46848 | 3.007123 |
| H | -3.54861 | 2.585343 | 1.481251 |
| H | -2.79604 | 1.336683 | 2.475922 |
| H | -1.84457 | 2.709176 | 1.912133 |
| H | -5.36298 | 0.466886 | -0.34382 |
| H | 1.784841 | 1.98834 | 1.765937 |
| H | 0.139 | 2.558358 | 1.967008 |
| H | 0.637213 | 1.028861 | 2.691092 |
| H | 2.890741 | 1.058865 | -0.48953 |
| H | 3.309903 | 0.386632 | 1.064827 |
| H | 3.635709 | -1.86696 | -0.07468 |
| H | 3.232958 | -1.11198 | -1.60413 |
| H | 5.382042 | -0.09042 | -2.98783 |
| H | 6.015924 | -0.58676 | 1.263983 |
| H | 8.247611 | 0.254165 | -0.08354 |
| H | -0.30377 | -2.57002 | 2.289169 |
| H | -1.41015 | -2.47354 | -0.00143 |
| C | -1.22162 | -0.73548 | 1.613076 |
| H | -1.06666 | -0.28112 | -0.44575 |
| H | -1.06877 | -0.28284 | 2.597258 |
| H | -2.2366 | -1.14868 | 1.614876 |
| C | -6.46396 | -1.52493 | -1.45539 |
| O | -6.61674 | -0.42912 | -0.94263 |
| O | -5.32781 | -2.15046 | -1.60371 |
| H | -7.30805 | -2.10143 | -1.85242 |
| H | -4.57836 | -1.59538 | -1.22805 |

Conf9 (-1269.088515 Hartree)

| Symbol | X | Y | Z |
| --- | --- | --- | --- |
| C | 0.322514 | -1.54724 | -2.75033 |
| C | 1.572073 | -0.79893 | -2.30154 |
| C | -0.88332 | -1.20328 | -1.87822 |
| C | -0.66476 | -1.50446 | -0.38149 |
| C | 0.660297 | -0.80174 | 0.058558 |
| C | 1.923745 | -1.07858 | -0.8173 |
| C | -1.80499 | -0.82195 | 0.46928 |
| C | -1.51392 | -1.00567 | 1.945153 |
| C | -0.21014 | -0.3944 | 2.393442 |
| C | -2.26222 | -1.68411 | 2.815079 |
| C | 2.534599 | -2.47413 | -0.6571 |
| C | 2.992303 | -0.05554 | -0.43109 |
| O | 4.146073 | -0.36859 | -0.15351 |
| O | 2.581597 | 1.195753 | -0.44572 |
| C | -0.70783 | -3.0221 | -0.14949 |
| C | -3.23405 | -1.20583 | 0.072178 |
| C | -4.29562 | -0.20592 | 0.571711 |
| C | -4.23632 | 1.127008 | -0.10881 |
| C | -3.77857 | 2.301067 | 0.4038 |
| C | -4.6404 | 1.436489 | -1.45398 |
| C | -4.39677 | 2.758128 | -1.63686 |
| O | -3.86958 | 3.303641 | -0.51162 |
| H | 0.501503 | -2.62801 | -2.74793 |
| H | 0.10426 | -1.28293 | -3.79057 |
| H | 1.400869 | 0.27548 | -2.42444 |
| H | 2.428369 | -1.06277 | -2.9324 |
| H | -1.75611 | -1.75043 | -2.24713 |
| H | -1.10962 | -0.13454 | -1.99263 |
| H | -1.69957 | 0.25401 | 0.273964 |
| O | -0.31898 | 1.022177 | 2.233693 |
| H | -3.18876 | -2.17425 | 2.537766 |
| H | -1.96067 | -1.77162 | 3.854906 |
| H | 3.477575 | -2.53196 | -1.20387 |
| H | 2.749739 | -2.70579 | 0.388006 |
| H | 1.872748 | -3.2459 | -1.04541 |
| H | 3.331776 | 1.812034 | -0.20889 |
| H | -0.05368 | -3.56799 | -0.82923 |
| H | -0.43558 | -3.29763 | 0.871491 |
| H | -1.72244 | -3.39157 | -0.32476 |
| H | -3.31153 | -1.26185 | -1.01661 |
| H | -3.48624 | -2.20365 | 0.444788 |
| H | -5.28734 | -0.64325 | 0.40946 |
| H | -4.19108 | -0.06482 | 1.6508 |
| H | -3.36345 | 2.571449 | 1.361702 |
| H | -5.06386 | 0.75788 | -2.18006 |
| H | -4.54014 | 3.433072 | -2.46539 |
| H | -0.04106 | -0.63392 | 3.451749 |
| H | 0.558408 | 1.398466 | 2.354061 |
| C | 0.933473 | -0.96812 | 1.554482 |
| H | 0.458034 | 0.26461 | -0.09254 |
| H | 1.068173 | -2.02053 | 1.823256 |
| H | 1.865498 | -0.4603 | 1.837012 |
| C | 5.684996 | 2.492628 | 0.45504 |
| O | 4.54799 | 2.858722 | 0.20835 |
| O | 6.111268 | 1.25932 | 0.453659 |
| H | 6.479142 | 3.204507 | 0.710792 |
| H | 5.35746 | 0.634005 | 0.218196 |

**Atomic coordinates**

(4*R*,5*R*,7*R*,9*R*,10*R*) – labdane (**6**)

B3PW91/PCM(CHCl_3_)/6-311G(d,p) level

Conf1 (-1344.331643 Hartree)

| Symbol | X | Y | Z |
| --- | --- | --- | --- |
| C | -0.95795 | 2.896445 | -1.13472 |
| C | 0.176329 | 1.895867 | -0.92432 |
| C | 0.021028 | 1.058653 | 0.36142 |
| C | -1.39994 | 0.407512 | 0.345345 |
| C | 1.035104 | -0.14823 | 0.336836 |
| C | 0.805943 | -1.01856 | 1.555756 |
| C | -0.5827 | -1.60868 | 1.632519 |
| C | -1.6318 | -0.5069 | 1.55193 |
| C | -2.60718 | 1.370719 | 0.080725 |
| C | -2.31125 | 2.200467 | -1.19149 |
| C | 0.319302 | 1.937365 | 1.585749 |
| C | -2.97673 | 2.285746 | 1.252923 |
| C | -3.78304 | 0.455596 | -0.26823 |
| O | -0.82719 | -2.50121 | 0.541701 |
| C | 1.682624 | -1.24884 | 2.534492 |
| C | 2.502877 | 0.237017 | 0.119031 |
| C | 3.346817 | -0.94801 | -0.3501 |
| C | 4.79855 | -0.66582 | -0.51691 |
| C | 5.498565 | 0.454839 | -0.30363 |
| C | 6.912678 | 0.208381 | -0.61485 |
| O | 7.862736 | 0.944736 | -0.55672 |
| O | 7.035948 | -1.09379 | -1.0208 |
| C | 5.755752 | -1.71888 | -0.99082 |
| O | -3.76092 | -0.27094 | -1.25842 |
| O | -4.80319 | 0.497574 | 0.558415 |
| H | -0.79399 | 3.436285 | -2.07333 |
| H | -0.94723 | 3.65752 | -0.34695 |
| H | 1.12852 | 2.43492 | -0.90843 |
| H | 0.210533 | 1.215668 | -1.78658 |
| H | -1.39212 | -0.24617 | -0.53534 |
| H | 0.730221 | -0.75411 | -0.52869 |
| H | -0.69599 | -2.14734 | 2.582847 |
| H | -1.61295 | 0.058185 | 2.488368 |
| H | -2.61506 | -0.98563 | 1.492968 |
| H | -3.11463 | 2.930528 | -1.34242 |
| H | -2.32654 | 1.523598 | -2.05128 |
| H | 0.085132 | 1.438476 | 2.528152 |
| H | 1.383403 | 2.190277 | 1.609664 |
| H | -0.22611 | 2.880588 | 1.562896 |
| H | -2.19912 | 3.025887 | 1.434916 |
| H | -3.90047 | 2.82543 | 1.032004 |
| H | -3.13708 | 1.729302 | 2.177472 |
| H | -0.1479 | -3.18168 | 0.571293 |
| H | 2.683906 | -0.83159 | 2.545848 |
| H | 1.410382 | -1.87065 | 3.382817 |
| H | 2.568528 | 1.020897 | -0.63899 |
| H | 2.933147 | 0.658646 | 1.03304 |
| H | 3.240634 | -1.79168 | 0.343419 |
| H | 2.958325 | -1.3194 | -1.30854 |
| H | 5.146302 | 1.416035 | 0.040125 |
| H | 5.794545 | -2.57862 | -0.3138 |
| H | 5.511091 | -2.07925 | -1.99539 |
| H | -5.51771 | -0.13577 | 0.258237 |
| C | -6.63135 | -1.9005 | -1.17365 |
| O | -6.71777 | -1.17927 | -0.1934 |
| O | -5.62214 | -1.95749 | -1.99793 |
| H | -7.43494 | -2.59224 | -1.45416 |
| H | -4.9046 | -1.31014 | -1.711 |

Conf2 (-1344.331291 Hartree)

| Symbol | X | Y | Z |
| --- | --- | --- | --- |
| C | -0.94887 | 3.062272 | -0.41075 |
| C | 0.191852 | 2.047892 | -0.44478 |
| C | 0.03661 | 0.915135 | 0.5901 |
| C | -1.37978 | 0.280782 | 0.402961 |
| C | 1.055723 | -0.24499 | 0.267269 |
| C | 0.825831 | -1.3978 | 1.223604 |
| C | -0.56431 | -1.98646 | 1.152781 |
| C | -1.61285 | -0.89858 | 1.350174 |
| C | -2.59289 | 1.270479 | 0.366194 |
| C | -2.29566 | 2.395097 | -0.65591 |
| C | 0.327919 | 1.459935 | 1.995937 |
| C | -2.98936 | 1.860527 | 1.723171 |
| C | -3.78891 | 0.478405 | -0.16443 |
| O | -0.81103 | -2.57729 | -0.12635 |
| C | 1.708228 | -1.87527 | 2.102421 |
| C | 2.521046 | 0.193441 | 0.160901 |
| C | 3.377556 | -0.81874 | -0.62325 |
| C | 4.818344 | -0.44218 | -0.68253 |
| C | 5.881156 | -1.08458 | -0.18474 |
| C | 7.09911 | -0.32662 | -0.49913 |
| O | 8.250808 | -0.55501 | -0.23573 |
| O | 6.729767 | 0.788878 | -1.20324 |
| C | 5.313438 | 0.799565 | -1.36291 |
| O | -4.82703 | 0.331038 | 0.473528 |
| O | -3.61512 | -0.0286 | -1.36589 |
| H | -0.781 | 3.820736 | -1.1828 |
| H | -0.95216 | 3.602743 | 0.541967 |
| H | 1.137684 | 2.574762 | -0.28599 |
| H | 0.238955 | 1.604399 | -1.44924 |
| H | -1.3535 | -0.14456 | -0.60683 |
| H | 0.758833 | -0.61206 | -0.72575 |
| H | -0.67666 | -2.74564 | 1.938252 |
| H | -1.59006 | -0.57963 | 2.396249 |
| H | -2.59765 | -1.34657 | 1.179148 |
| H | -3.10414 | 3.134057 | -0.62313 |
| H | -2.29571 | 1.964656 | -1.66187 |
| H | 0.134284 | 0.724609 | 2.779299 |
| H | 1.382283 | 1.742316 | 2.072387 |
| H | -0.25331 | 2.35269 | 2.225737 |
| H | -2.22928 | 2.543611 | 2.098602 |
| H | -3.92288 | 2.418917 | 1.626095 |
| H | -3.15181 | 1.085246 | 2.473786 |
| H | -0.13142 | -3.24276 | -0.2706 |
| H | 2.711412 | -1.47737 | 2.21099 |
| H | 1.440964 | -2.695 | 2.763464 |
| H | 2.573142 | 1.156381 | -0.3515 |
| H | 2.957423 | 0.349903 | 1.15229 |
| H | 3.285755 | -1.81472 | -0.18507 |
| H | 2.987732 | -0.8827 | -1.64754 |
| H | 5.901912 | -2.016 | 0.362884 |
| H | 5.074033 | 0.807384 | -2.43162 |
| H | 4.916818 | 1.714777 | -0.91251 |
| H | -4.42467 | -0.54144 | -1.64951 |
| C | -6.75051 | -1.53484 | -1.46623 |
| O | -5.75088 | -1.3991 | -2.15153 |
| O | -6.93864 | -1.03429 | -0.27575 |
| H | -7.614 | -2.11631 | -1.8115 |
| H | -6.1281 | -0.50835 | 0.008741 |

Conf3 (-1344.330784 Hartree)

| Symbol | X | Y | Z |
| --- | --- | --- | --- |
| C | -1.03812 | 2.959399 | -0.61797 |
| C | 0.12535 | 1.971058 | -0.60093 |
| C | 0.040819 | 0.947374 | 0.548574 |
| C | -1.37032 | 0.276527 | 0.495333 |
| C | 1.067534 | -0.22441 | 0.29286 |
| C | 0.885951 | -1.3101 | 1.335122 |
| C | -0.49856 | -1.91226 | 1.360973 |
| C | -1.52356 | -0.79892 | 1.572522 |
| C | -2.60547 | 1.21175 | 0.394757 |
| C | -2.37635 | 2.243297 | -0.74422 |
| C | 0.385794 | 1.636708 | 1.876194 |
| C | -2.99121 | 1.919929 | 1.701553 |
| C | -3.79 | 0.37898 | -0.10211 |
| O | -0.72004 | -2.59051 | 0.121025 |
| C | 1.799439 | -1.70374 | 2.222262 |
| C | 2.52268 | 0.227058 | 0.118416 |
| C | 3.373931 | -0.82328 | -0.61983 |
| C | 4.812974 | -0.44762 | -0.71302 |
| C | 5.884574 | -1.07549 | -0.21529 |
| C | 7.096276 | -0.32609 | -0.57044 |
| O | 8.252611 | -0.5463 | -0.32009 |
| O | 6.714608 | 0.769908 | -1.29854 |
| C | 5.295649 | 0.775408 | -1.43481 |
| O | -3.66145 | -0.7004 | -0.67785 |
| O | -4.96005 | 0.947557 | 0.077349 |
| H | -0.92127 | 3.644874 | -1.464 |
| H | -1.02312 | 3.587036 | 0.279719 |
| H | 1.063673 | 2.530415 | -0.53906 |
| H | 0.141341 | 1.429337 | -1.55688 |
| H | -1.37205 | -0.25474 | -0.46312 |
| H | 0.753894 | -0.67272 | -0.6605 |
| H | -0.56453 | -2.6344 | 2.185058 |
| H | -1.39344 | -0.3852 | 2.576993 |
| H | -2.52766 | -1.23735 | 1.545042 |
| H | -3.20509 | 2.957504 | -0.75393 |
| H | -2.39722 | 1.71489 | -1.70601 |
| H | 1.425815 | 1.975781 | 1.858073 |
| H | -0.22866 | 2.518054 | 2.060325 |
| H | 0.280253 | 0.969384 | 2.733827 |
| H | -2.21555 | 2.616861 | 2.015236 |
| H | -3.9146 | 2.484443 | 1.568586 |
| H | -3.1485 | 1.207918 | 2.515432 |
| H | -1.65684 | -2.5197 | -0.09005 |
| H | 2.794008 | -1.27489 | 2.277355 |
| H | 1.57079 | -2.48657 | 2.939578 |
| H | 2.548215 | 1.156062 | -0.45567 |
| H | 2.982416 | 0.454306 | 1.085529 |
| H | 3.287408 | -1.79489 | -0.12923 |
| H | 2.974354 | -0.94259 | -1.63533 |
| H | 5.914683 | -1.99145 | 0.35735 |
| H | 5.038455 | 0.754497 | -2.4992 |
| H | 4.905614 | 1.702113 | -1.00239 |
| H | -5.68769 | 0.385913 | -0.3185 |
| C | -6.74464 | -1.573 | -1.52099 |
| O | -6.92248 | -0.51099 | -0.94837 |
| O | -5.59187 | -2.15215 | -1.72059 |
| H | -7.57722 | -2.15487 | -1.93392 |
| H | -4.85466 | -1.59238 | -1.32765 |

Conf4 (-1344.330760 Hartree)

| Symbol | X | Y | Z |
| --- | --- | --- | --- |
| C | -0.84504 | 3.040562 | -0.39591 |
| C | 0.273041 | 1.999932 | -0.38193 |
| C | 0.052661 | 0.876277 | 0.649822 |
| C | -1.37055 | 0.274215 | 0.415671 |
| C | 1.053381 | -0.31037 | 0.365227 |
| C | 0.765088 | -1.45071 | 1.319319 |
| C | -0.63459 | -2.00902 | 1.202428 |
| C | -1.66476 | -0.89627 | 1.356856 |
| C | -2.55663 | 1.293032 | 0.335611 |
| C | -2.1982 | 2.402592 | -0.68329 |
| C | 0.307167 | 1.419081 | 2.063583 |
| C | -2.98019 | 1.901519 | 1.676168 |
| C | -3.75546 | 0.527766 | -0.22697 |
| O | -0.84878 | -2.60121 | -0.08166 |
| C | 1.604237 | -1.94041 | 2.233067 |
| C | 2.530301 | 0.093608 | 0.298058 |
| C | 3.388872 | -0.94242 | -0.45249 |
| C | 4.817793 | -0.53567 | -0.56764 |
| C | 5.515746 | -0.20322 | -1.65966 |
| C | 6.893616 | 0.127449 | -1.27441 |
| O | 7.830758 | 0.47318 | -1.94527 |
| O | 6.996937 | -0.02 | 0.084008 |
| C | 5.739038 | -0.43348 | 0.611411 |
| O | -4.81535 | 0.408942 | 0.380528 |
| O | -3.55845 | 0.010268 | -1.42022 |
| H | -0.63221 | 3.788389 | -1.16708 |
| H | -0.87004 | 3.588867 | 0.5521 |
| H | 1.224173 | 2.505509 | -0.1894 |
| H | 0.348798 | 1.551348 | -1.38223 |
| H | -1.32003 | -0.15509 | -0.59153 |
| H | 0.777031 | -0.6753 | -0.63428 |
| H | -0.79164 | -2.76101 | 1.987197 |
| H | -1.6743 | -0.57464 | 2.40232 |
| H | -2.65205 | -1.32305 | 1.149996 |
| H | -2.98954 | 3.16052 | -0.68377 |
| H | -2.17358 | 1.963987 | -1.68538 |
| H | 0.049068 | 0.699233 | 2.842746 |
| H | 1.367987 | 1.658791 | 2.183354 |
| H | -0.24491 | 2.33758 | 2.262084 |
| H | -2.21068 | 2.558551 | 2.077677 |
| H | -3.8898 | 2.491535 | 1.544832 |
| H | -3.19722 | 1.135084 | 2.422087 |
| H | -0.17969 | -3.28251 | -0.19879 |
| H | 2.61042 | -1.56158 | 2.376204 |
| H | 1.296529 | -2.74935 | 2.889712 |
| H | 2.626616 | 1.043836 | -0.23153 |
| H | 2.932878 | 0.260041 | 1.301852 |
| H | 3.332237 | -1.90946 | 0.060017 |
| H | 2.979466 | -1.08822 | -1.45625 |
| H | 5.175451 | -0.17083 | -2.68488 |
| H | 5.406445 | 0.306654 | 1.345979 |
| H | 5.864033 | -1.39516 | 1.120244 |
| H | -4.37238 | -0.48206 | -1.72666 |
| C | -6.72885 | -1.41136 | -1.61168 |
| O | -5.7062 | -1.30433 | -2.26745 |
| O | -6.93809 | -0.90337 | -0.42791 |
| H | -7.5973 | -1.96993 | -1.98145 |
| H | -6.12244 | -0.39827 | -0.12081 |

Conf5 (-1344.330556 Hartree)

| Symbol | X | Y | Z |
| --- | --- | --- | --- |
| C | -0.96512 | 3.039459 | -0.46363 |
| C | 0.180885 | 2.030768 | -0.49458 |
| C | 0.045264 | 0.91267 | 0.559289 |
| C | -1.37101 | 0.272447 | 0.39634 |
| C | 1.066981 | -0.24639 | 0.238069 |
| C | 0.840459 | -1.40306 | 1.190864 |
| C | -0.54126 | -2.00161 | 1.104815 |
| C | -1.58257 | -0.90623 | 1.348251 |
| C | -2.58868 | 1.252999 | 0.367037 |
| C | -2.31309 | 2.36313 | -0.67866 |
| C | 0.349563 | 1.477517 | 1.954058 |
| C | -2.96608 | 1.863551 | 1.720203 |
| C | -3.7941 | 0.458995 | -0.13893 |
| O | -0.6812 | -2.57765 | -0.19576 |
| C | 1.710039 | -1.85566 | 2.09351 |
| C | 2.531248 | 0.196938 | 0.138783 |
| C | 3.396176 | -0.82013 | -0.6299 |
| C | 4.837186 | -0.44304 | -0.6741 |
| C | 5.893194 | -1.0756 | -0.14961 |
| C | 7.114503 | -0.32027 | -0.4562 |
| O | 8.262424 | -0.5418 | -0.17057 |
| O | 6.754846 | 0.783405 | -1.18371 |
| C | 5.341155 | 0.788434 | -1.36643 |
| O | -4.85658 | 0.396964 | 0.471589 |
| O | -3.60422 | -0.14396 | -1.29432 |
| H | -0.81178 | 3.784511 | -1.25163 |
| H | -0.95871 | 3.597787 | 0.478746 |
| H | 1.125579 | 2.565164 | -0.3561 |
| H | 0.218658 | 1.571105 | -1.49206 |
| H | -1.35302 | -0.15758 | -0.61158 |
| H | 0.775735 | -0.6147 | -0.75555 |
| H | -0.64698 | -2.78262 | 1.869106 |
| H | -1.52267 | -0.5933 | 2.395392 |
| H | -2.5842 | -1.33734 | 1.218501 |
| H | -3.12414 | 3.099038 | -0.64537 |
| H | -2.32594 | 1.915605 | -1.67755 |
| H | 0.180772 | 0.746737 | 2.747509 |
| H | 1.400143 | 1.777554 | 2.010841 |
| H | -0.24227 | 2.363057 | 2.18433 |
| H | -2.21096 | 2.568546 | 2.063259 |
| H | -3.91219 | 2.40141 | 1.633956 |
| H | -3.09412 | 1.10102 | 2.490839 |
| H | -1.60422 | -2.82497 | -0.3086 |
| H | 2.699179 | -1.43056 | 2.224581 |
| H | 1.449641 | -2.68296 | 2.747097 |
| H | 2.583957 | 1.15661 | -0.3802 |
| H | 2.960323 | 0.362144 | 1.131886 |
| H | 3.297756 | -1.8127 | -0.18598 |
| H | 3.017592 | -0.89176 | -1.6577 |
| H | 5.906187 | -1.99847 | 0.412518 |
| H | 5.119139 | 0.778057 | -2.43888 |
| H | 4.935472 | 1.710199 | -0.93789 |
| H | -4.42902 | -0.63757 | -1.56943 |
| C | -6.80422 | -1.50653 | -1.40565 |
| O | -5.76634 | -1.4875 | -2.04643 |
| O | -7.01639 | -0.89723 | -0.27148 |
| H | -7.68438 | -2.06469 | -1.74685 |
| H | -6.18904 | -0.3976 | 0.010959 |

Conf6 (-1344.330435 Hartree)

| Symbol | X | Y | Z |
| --- | --- | --- | --- |
| C | -0.94864 | 2.897889 | -0.98673 |
| C | 0.195275 | 1.897 | -0.83296 |
| C | 0.04911 | 0.982149 | 0.399512 |
| C | -1.36715 | 0.324841 | 0.340119 |
| C | 1.073478 | -0.21382 | 0.297079 |
| C | 0.837161 | -1.18461 | 1.436138 |
| C | -0.54332 | -1.79186 | 1.442072 |
| C | -1.5889 | -0.6748 | 1.476315 |
| C | -2.57897 | 1.29166 | 0.143681 |
| C | -2.29906 | 2.198098 | -1.08278 |
| C | 0.344273 | 1.782084 | 1.676724 |
| C | -2.94646 | 2.134887 | 1.368359 |
| C | -3.79625 | 0.439874 | -0.21915 |
| O | -0.66678 | -2.58949 | 0.261867 |
| C | 1.697443 | -1.47228 | 2.412612 |
| C | 2.539578 | 0.205233 | 0.13753 |
| C | 3.406337 | -0.92207 | -0.42293 |
| C | 4.854613 | -0.6019 | -0.54418 |
| C | 5.533711 | 0.500917 | -0.20558 |
| C | 6.953893 | 0.313051 | -0.52793 |
| O | 7.890251 | 1.055238 | -0.38259 |
| O | 7.103411 | -0.93542 | -1.07013 |
| C | 5.833886 | -1.58165 | -1.11963 |
| O | -4.87746 | 0.542723 | 0.351646 |
| O | -3.59747 | -0.39985 | -1.21441 |
| H | -0.78993 | 3.491213 | -1.89337 |
| H | -0.94474 | 3.613151 | -0.15731 |
| H | 1.140982 | 2.445094 | -0.78397 |
| H | 0.238743 | 1.270282 | -1.73434 |
| H | -1.34664 | -0.2678 | -0.58136 |
| H | 0.791006 | -0.75234 | -0.61846 |
| H | -0.65724 | -2.42647 | 2.330641 |
| H | -1.54665 | -0.18744 | 2.455442 |
| H | -2.58687 | -1.12771 | 1.405653 |
| H | -3.10737 | 2.930881 | -1.18354 |
| H | -2.31146 | 1.57945 | -1.98604 |
| H | 0.154358 | 1.204842 | 2.583903 |
| H | 1.398499 | 2.073447 | 1.694506 |
| H | -0.23605 | 2.702075 | 1.73759 |
| H | -2.18074 | 2.877359 | 1.584779 |
| H | -3.88458 | 2.66279 | 1.187706 |
| H | -3.08583 | 1.520164 | 2.259872 |
| H | -1.59094 | -2.84264 | 0.174846 |
| H | 2.686032 | -1.03094 | 2.477207 |
| H | 1.428644 | -2.17229 | 3.198175 |
| H | 2.607834 | 1.055106 | -0.54592 |
| H | 2.951751 | 0.548011 | 1.091846 |
| H | 3.303717 | -1.82568 | 0.190962 |
| H | 3.035514 | -1.21283 | -1.41543 |
| H | 5.162431 | 1.414178 | 0.235345 |
| H | 5.882503 | -2.509 | -0.53951 |
| H | 5.603115 | -1.83516 | -2.15953 |
| H | -4.43421 | -0.90982 | -1.41299 |
| C | -6.8514 | -1.62694 | -1.18097 |
| O | -5.79227 | -1.78509 | -1.76524 |
| O | -7.07176 | -0.78722 | -0.20691 |
| H | -7.74631 | -2.20591 | -1.43902 |
| H | -6.23064 | -0.27561 | 0.004355 |

Conf7 (-1344.330186 Hartree)

| Symbol | X | Y | Z |
| --- | --- | --- | --- |
| C | -0.8593 | 3.012339 | -0.45373 |
| C | 0.264305 | 1.977696 | -0.42972 |
| C | 0.061772 | 0.873871 | 0.627196 |
| C | -1.36158 | 0.264619 | 0.418685 |
| C | 1.064791 | -0.31371 | 0.35172 |
| C | 0.780083 | -1.45215 | 1.30949 |
| C | -0.61192 | -2.02029 | 1.184144 |
| C | -1.63648 | -0.89796 | 1.373902 |
| C | -2.55336 | 1.272524 | 0.334762 |
| C | -2.21324 | 2.363258 | -0.71269 |
| C | 0.329217 | 1.444173 | 2.027253 |
| C | -2.96396 | 1.907106 | 1.666885 |
| C | -3.75994 | 0.50394 | -0.20649 |
| O | -0.71875 | -2.61312 | -0.11185 |
| C | 1.608671 | -1.91372 | 2.24561 |
| C | 2.540417 | 0.095157 | 0.287729 |
| C | 3.405255 | -0.94641 | -0.44778 |
| C | 4.833274 | -0.53611 | -0.55897 |
| C | 5.534782 | -0.20516 | -1.64939 |
| C | 6.909388 | 0.132663 | -1.2597 |
| O | 7.848 | 0.480357 | -1.92793 |
| O | 7.007919 | -0.00928 | 0.099554 |
| C | 5.749598 | -0.42677 | 0.62316 |
| O | -4.84897 | 0.487428 | 0.358718 |
| O | -3.54016 | -0.12771 | -1.34088 |
| H | -0.65898 | 3.744337 | -1.24324 |
| H | -0.87708 | 3.581331 | 0.482215 |
| H | 1.214814 | 2.491899 | -0.25894 |
| H | 0.331204 | 1.508145 | -1.42092 |
| H | -1.31643 | -0.17765 | -0.58291 |
| H | 0.793795 | -0.68623 | -0.6459 |
| H | -0.76192 | -2.78688 | 1.955646 |
| H | -1.61183 | -0.57529 | 2.419538 |
| H | -2.64139 | -1.30852 | 1.207999 |
| H | -3.00815 | 3.117173 | -0.71945 |
| H | -2.19765 | 1.902918 | -1.70574 |
| H | 1.387217 | 1.704726 | 2.125428 |
| H | -0.23614 | 2.355519 | 2.220764 |
| H | 0.097615 | 0.73163 | 2.821413 |
| H | -2.19464 | 2.579105 | 2.042648 |
| H | -3.88081 | 2.485238 | 1.536261 |
| H | -3.16138 | 1.154872 | 2.433037 |
| H | -1.64327 | -2.83741 | -0.25604 |
| H | 2.602251 | -1.50892 | 2.404726 |
| H | 1.308535 | -2.72669 | 2.90007 |
| H | 2.63591 | 1.040858 | -0.25049 |
| H | 2.938249 | 0.272845 | 1.291437 |
| H | 3.34656 | -1.90797 | 0.07433 |
| H | 3.001251 | -1.10435 | -1.45171 |
| H | 5.198359 | -0.17857 | -2.67605 |
| H | 5.876474 | -1.38683 | 1.134573 |
| H | 5.411228 | 0.31359 | 1.354889 |
| H | -4.36807 | -0.59973 | -1.6436 |
| C | -6.77504 | -1.38356 | -1.57358 |
| O | -5.70946 | -1.41278 | -2.16682 |
| O | -7.01639 | -0.74512 | -0.46156 |
| H | -7.65806 | -1.91776 | -1.94446 |
| H | -6.18553 | -0.26868 | -0.15034 |

Conf8 (-1344.330005 Hartree)

| Symbol | X | Y | Z |
| --- | --- | --- | --- |
| C | -0.95546 | 3.038341 | -0.61103 |
| C | 0.177383 | 2.015046 | -0.57205 |
| C | 0.023351 | 0.970611 | 0.550576 |
| C | -1.3995 | 0.335635 | 0.429457 |
| C | 1.031438 | -0.21945 | 0.308252 |
| C | 0.802677 | -1.29819 | 1.347075 |
| C | -0.59444 | -1.87457 | 1.339158 |
| C | -1.63014 | -0.76404 | 1.468051 |
| C | -2.60716 | 1.329568 | 0.324043 |
| C | -2.30858 | 2.363227 | -0.78723 |
| C | 0.333196 | 1.62053 | 1.906676 |
| C | -2.98741 | 2.031333 | 1.632477 |
| C | -3.77839 | 0.481993 | -0.17915 |
| O | -0.86574 | -2.55617 | 0.110916 |
| C | 1.69414 | -1.7254 | 2.24253 |
| C | 2.498786 | 0.201574 | 0.160989 |
| C | 3.341497 | -0.85953 | -0.57115 |
| C | 4.783298 | -0.49454 | -0.66897 |
| C | 5.851199 | -1.12479 | -0.16668 |
| C | 7.067713 | -0.38776 | -0.53216 |
| O | 8.222728 | -0.61426 | -0.28188 |
| O | 6.692294 | 0.702967 | -1.27085 |
| C | 5.273218 | 0.717735 | -1.40424 |
| O | -3.75476 | -0.058 | -1.28203 |
| O | -4.79463 | 0.368098 | 0.645663 |
| H | -0.7896 | 3.728935 | -1.44467 |
| H | -0.94486 | 3.656299 | 0.293747 |
| H | 1.128215 | 2.545631 | -0.46419 |
| H | 0.212349 | 1.491955 | -1.53776 |
| H | -1.39237 | -0.16651 | -0.54554 |
| H | 0.724662 | -0.65507 | -0.65365 |
| H | -0.70286 | -2.57216 | 2.180274 |
| H | -1.58958 | -0.36632 | 2.486151 |
| H | -2.62018 | -1.21545 | 1.345365 |
| H | -3.11147 | 3.108771 | -0.81183 |
| H | -2.32337 | 1.843455 | -1.7501 |
| H | -0.24527 | 2.528763 | 2.074129 |
| H | 0.149639 | 0.947246 | 2.746212 |
| H | 1.388562 | 1.906001 | 1.948217 |
| H | -2.22024 | 2.742632 | 1.934915 |
| H | -3.9194 | 2.586445 | 1.504334 |
| H | -3.13746 | 1.326016 | 2.451151 |
| H | -0.1941 | -3.23707 | 0.006752 |
| H | 2.703717 | -1.3343 | 2.306404 |
| H | 1.428448 | -2.49448 | 2.962381 |
| H | 2.552638 | 1.132417 | -0.40724 |
| H | 2.946848 | 0.413392 | 1.136944 |
| H | 3.249875 | -1.82801 | -0.07486 |
| H | 2.940517 | -0.98067 | -1.58602 |
| H | 5.876391 | -2.03489 | 0.415481 |
| H | 5.013492 | 0.688254 | -2.4678 |
| H | 4.891277 | 1.651757 | -0.98049 |
| H | -5.50392 | -0.21161 | 0.242325 |
| C | -6.60158 | -1.72017 | -1.46927 |
| O | -6.69186 | -1.17929 | -0.3794 |
| O | -5.59793 | -1.61815 | -2.29606 |
| H | -7.3963 | -2.36694 | -1.86048 |
| H | -4.88786 | -1.01902 | -1.90522 |

Conf9 (-1344.329984 Hartree)

| Symbol | X | Y | Z |
| --- | --- | --- | --- |
| C | 0.596471 | -1.59784 | -2.79766 |
| C | -0.61594 | -1.38569 | -1.89386 |
| C | -0.33657 | -1.6914 | -0.40855 |
| C | 0.924239 | -0.87338 | 0.021949 |
| C | -1.51798 | -1.13816 | 0.479764 |
| C | -1.18355 | -1.34004 | 1.943543 |
| C | 0.078902 | -0.63809 | 2.38842 |
| C | 1.250441 | -1.05221 | 1.506869 |
| C | 2.188541 | -1.00793 | -0.89183 |
| C | 1.776887 | -0.7401 | -2.36051 |
| C | -0.22914 | -3.21019 | -0.20565 |
| C | 2.937408 | -2.33886 | -0.77412 |
| C | 3.150335 | 0.107119 | -0.47783 |
| O | -0.04734 | 0.7817 | 2.275516 |
| C | -1.86402 | -2.09269 | 2.809582 |
| C | -2.91079 | -1.64657 | 0.09005 |
| C | -4.06385 | -0.7837 | 0.641727 |
| C | -4.14312 | 0.582116 | 0.047645 |
| C | -4.05139 | 1.774132 | 0.648219 |
| C | -4.22911 | 2.837462 | -0.34946 |
| O | -4.22046 | 4.034026 | -0.22486 |
| O | -4.42997 | 2.241707 | -1.5664 |
| C | -4.39248 | 0.825397 | -1.41143 |
| O | 4.281764 | -0.11104 | -0.05459 |
| O | 2.665252 | 1.321413 | -0.61811 |
| H | 0.3285 | -1.33761 | -3.82718 |
| H | 0.875193 | -2.65675 | -2.82118 |
| H | -1.44061 | -2.00655 | -2.25764 |
| H | -0.94019 | -0.33956 | -1.98238 |
| H | 0.62046 | 0.173156 | -0.09627 |
| H | -1.50899 | -0.05106 | 0.315924 |
| H | 0.295381 | -0.9084 | 3.430615 |
| H | 1.507293 | -2.09058 | 1.736279 |
| H | 2.115717 | -0.44364 | 1.790547 |
| H | 2.639795 | -0.91031 | -3.01423 |
| H | 1.501544 | 0.313885 | -2.46179 |
| H | 0.080544 | -3.47584 | 0.806891 |
| H | -1.20377 | -3.67648 | -0.37826 |
| H | 0.46817 | -3.67707 | -0.90101 |
| H | 2.361899 | -3.15776 | -1.20215 |
| H | 3.885621 | -2.28031 | -1.31277 |
| H | 3.168961 | -2.58909 | 0.262422 |
| H | -0.80358 | 1.046956 | 2.807468 |
| H | -2.76098 | -2.64098 | 2.543392 |
| H | -1.52668 | -2.19165 | 3.837535 |
| H | -2.99998 | -1.68726 | -0.99791 |
| H | -3.05979 | -2.67318 | 0.436541 |
| H | -5.00825 | -1.30143 | 0.430643 |
| H | -3.98652 | -0.69519 | 1.727373 |
| H | -3.88299 | 1.983155 | 1.694949 |
| H | -3.5996 | 0.423889 | -2.04989 |
| H | -5.34861 | 0.404198 | -1.74024 |
| H | 3.337137 | 2.003127 | -0.33036 |
| C | 5.547044 | 2.887375 | 0.542679 |
| O | 4.435769 | 3.158845 | 0.120396 |
| O | 6.038845 | 1.68782 | 0.69367 |
| H | 6.257639 | 3.666603 | 0.84404 |
| H | 5.361834 | 0.99938 | 0.406422 |

Conf10 (-1344.329631 Hartree)

| Symbol | X | Y | Z |
| --- | --- | --- | --- |
| C | 0.635655 | -1.65912 | -2.78633 |
| C | -0.5807 | -1.42863 | -1.89275 |
| C | -0.31287 | -1.72361 | -0.40335 |
| C | 0.952677 | -0.91438 | 0.028782 |
| C | -1.49528 | -1.15238 | 0.472545 |
| C | -1.17236 | -1.34554 | 1.939952 |
| C | 0.092842 | -0.65058 | 2.387146 |
| C | 1.266415 | -1.08414 | 1.517678 |
| C | 2.223354 | -1.06392 | -0.87614 |
| C | 1.819571 | -0.80826 | -2.34753 |
| C | -0.21898 | -3.2415 | -0.18702 |
| C | 2.966204 | -2.39731 | -0.74095 |
| C | 3.139479 | 0.09469 | -0.47429 |
| O | -0.01912 | 0.769121 | 2.259731 |
| C | -1.86366 | -2.08729 | 2.806841 |
| C | -2.88983 | -1.65164 | 0.077411 |
| C | -4.03996 | -0.77969 | 0.620801 |
| C | -4.10268 | 0.588111 | 0.02936 |
| C | -4.00679 | 1.777701 | 0.634091 |
| C | -4.16691 | 2.845156 | -0.36214 |
| O | -4.14979 | 4.041281 | -0.23411 |
| O | -4.36155 | 2.254365 | -1.58249 |
| C | -4.33689 | 0.83737 | -1.43115 |
| O | 2.810169 | 1.264963 | -0.6468 |
| O | 4.286245 | -0.24739 | 0.068686 |
| H | 0.377164 | -1.40518 | -3.81983 |
| H | 0.904716 | -2.72092 | -2.79941 |
| H | -1.4087 | -2.04541 | -2.25591 |
| H | -0.89468 | -0.38041 | -1.99269 |
| H | 0.664584 | 0.135609 | -0.10332 |
| H | -1.47519 | -0.0668 | 0.29984 |
| H | 0.299846 | -0.91292 | 3.433303 |
| H | 1.510202 | -2.12334 | 1.757316 |
| H | 2.135618 | -0.48265 | 1.803382 |
| H | 2.68459 | -0.98754 | -2.99624 |
| H | 1.555186 | 0.248519 | -2.45065 |
| H | -1.19457 | -3.70255 | -0.36807 |
| H | 0.483679 | -3.71847 | -0.8701 |
| H | 0.076527 | -3.50121 | 0.83127 |
| H | 2.385811 | -3.21512 | -1.16536 |
| H | 3.915881 | -2.35483 | -1.27901 |
| H | 3.190168 | -2.64339 | 0.298047 |
| H | -0.77633 | 1.046796 | 2.783886 |
| H | -2.76242 | -2.63156 | 2.538463 |
| H | -1.53376 | -2.18124 | 3.837685 |
| H | -2.97367 | -1.69454 | -1.01088 |
| H | -3.04781 | -2.67621 | 0.425998 |
| H | -4.98681 | -1.28902 | 0.400368 |
| H | -3.97155 | -0.6939 | 1.707219 |
| H | -3.84622 | 1.982388 | 1.682912 |
| H | -3.54163 | 0.431226 | -2.06365 |
| H | -5.29343 | 0.424894 | -1.7697 |
| H | 4.81228 | 0.570169 | 0.306871 |
| C | 5.32514 | 3.038295 | 0.549049 |
| O | 5.710938 | 1.894014 | 0.723973 |
| O | 4.187466 | 3.392261 | 0.018826 |
| H | 5.934455 | 3.903454 | 0.837468 |
| H | 3.65474 | 2.5751 | -0.23379 |

Conf11 (-1344.329463 Hartree)

| Symbol | X | Y | Z |
| --- | --- | --- | --- |
| C | -0.90882 | 2.941327 | -0.56126 |
| C | 0.22321 | 1.91809 | -0.50631 |
| C | 0.064518 | 0.894995 | 0.634484 |
| C | -1.36117 | 0.264193 | 0.522768 |
| C | 1.066998 | -0.3054 | 0.412935 |
| C | 0.815531 | -1.38782 | 1.443243 |
| C | -0.58551 | -1.95087 | 1.413382 |
| C | -1.58655 | -0.8092 | 1.589397 |
| C | -2.5652 | 1.232997 | 0.37643 |
| C | -2.26148 | 2.266102 | -0.74425 |
| C | 0.377102 | 1.570136 | 1.977355 |
| C | -2.987 | 1.94236 | 1.671748 |
| C | -3.75175 | 0.436981 | -0.17253 |
| O | -0.77885 | -2.61953 | 0.163684 |
| C | 1.682827 | -1.80663 | 2.364598 |
| C | 2.538089 | 0.107853 | 0.289087 |
| C | 3.387812 | -0.95665 | -0.43138 |
| C | 4.808305 | -0.5406 | -0.6019 |
| C | 5.468597 | -0.22943 | -1.72349 |
| C | 6.852545 | 0.129576 | -1.38976 |
| O | 7.763934 | 0.470404 | -2.098 |
| O | 6.999579 | 0.019752 | -0.03195 |
| C | 5.763998 | -0.39577 | 0.544597 |
| O | -3.63686 | -0.65754 | -0.72193 |
| O | -4.90574 | 1.054766 | -0.06562 |
| H | -0.73875 | 3.627783 | -1.39732 |
| H | -0.90992 | 3.562978 | 0.340804 |
| H | 1.175275 | 2.44782 | -0.40862 |
| H | 0.257331 | 1.378684 | -1.46298 |
| H | -1.33887 | -0.26465 | -0.43683 |
| H | 0.773942 | -0.74155 | -0.5524 |
| H | -0.70339 | -2.67332 | 2.231429 |
| H | -1.48369 | -0.40193 | 2.599593 |
| H | -2.60012 | -1.21983 | 1.52096 |
| H | -3.0685 | 3.003766 | -0.78048 |
| H | -2.25994 | 1.745813 | -1.71079 |
| H | 0.226134 | 0.901853 | 2.827434 |
| H | 1.424401 | 1.885876 | 1.997625 |
| H | -0.22311 | 2.464624 | 2.144085 |
| H | -2.20074 | 2.6038 | 2.032034 |
| H | -3.88014 | 2.544208 | 1.501013 |
| H | -3.21222 | 1.22855 | 2.467946 |
| H | -1.70022 | -2.50551 | -0.09174 |
| H | 2.68452 | -1.40194 | 2.459727 |
| H | 1.40712 | -2.58372 | 3.071478 |
| H | 2.613591 | 1.032671 | -0.28732 |
| H | 2.963369 | 0.326244 | 1.273546 |
| H | 3.351025 | -1.89831 | 0.127867 |
| H | 2.954917 | -1.15451 | -1.41601 |
| H | 5.096869 | -0.22938 | -2.73827 |
| H | 5.444113 | 0.358774 | 1.270042 |
| H | 5.917168 | -1.3425 | 1.073301 |
| H | -5.63347 | 0.515442 | -0.49096 |
| C | -6.70655 | -1.42729 | -1.70585 |
| O | -6.86871 | -0.34394 | -1.16986 |
| O | -5.57054 | -2.05857 | -1.82939 |
| H | -7.54036 | -1.98546 | -2.14803 |
| H | -4.83192 | -1.5188 | -1.41244 |

Conf12 (-1344.328854 Hartree)

| Symbol | X | Y | Z |
| --- | --- | --- | --- |
| C | 0.582488 | -1.40755 | -2.81147 |
| C | -0.64249 | -1.21841 | -1.92018 |
| C | -0.41595 | -1.68406 | -0.46804 |
| C | 0.879132 | -0.98989 | 0.065275 |
| C | -1.58146 | -1.14692 | 0.450211 |
| C | -1.29012 | -1.47602 | 1.901528 |
| C | 0.000511 | -0.88525 | 2.416046 |
| C | 1.151194 | -1.35372 | 1.527531 |
| C | 2.149924 | -1.06319 | -0.82073 |
| C | 1.78797 | -0.64732 | -2.27378 |
| C | -0.40223 | -3.21872 | -0.41837 |
| C | 2.871799 | -2.41886 | -0.80747 |
| C | 3.115289 | 0.031138 | -0.35896 |
| O | -0.11614 | 0.540359 | 2.407592 |
| C | -2.02536 | -2.25721 | 2.692845 |
| C | -2.99238 | -1.54111 | 0.002886 |
| C | -4.09423 | -0.63502 | 0.597803 |
| C | -4.08822 | 0.744528 | 0.031372 |
| C | -3.62674 | 1.882948 | 0.562685 |
| C | -3.83034 | 2.98059 | -0.39195 |
| O | -3.55731 | 4.149552 | -0.309 |
| O | -4.43207 | 2.461538 | -1.50887 |
| C | -4.63639 | 1.063187 | -1.32731 |
| O | 2.76996 | 0.991907 | 0.326823 |
| O | 4.338973 | -0.10644 | -0.81565 |
| H | 0.357703 | -1.04791 | -3.82107 |
| H | 0.818776 | -2.47177 | -2.91775 |
| H | -1.48781 | -1.75629 | -2.36 |
| H | -0.91229 | -0.15341 | -1.91183 |
| H | 0.625333 | 0.076311 | 0.061983 |
| H | -1.51406 | -0.05308 | 0.374851 |
| H | 0.169559 | -1.22594 | 3.44582 |
| H | 1.277534 | -2.43277 | 1.65695 |
| H | 2.078107 | -0.8877 | 1.880109 |
| H | 2.661597 | -0.79045 | -2.91654 |
| H | 1.553874 | 0.424963 | -2.28523 |
| H | 0.314213 | -3.65469 | -1.1151 |
| H | -0.17612 | -3.60319 | 0.578345 |
| H | -1.38693 | -3.60667 | -0.6965 |
| H | 2.250142 | -3.19945 | -1.2437 |
| H | 3.794678 | -2.36316 | -1.38577 |
| H | 3.131527 | -2.72803 | 0.208022 |
| H | 0.74615 | 0.907371 | 2.185844 |
| H | -2.93827 | -2.74223 | 2.364713 |
| H | -1.72458 | -2.44027 | 3.720392 |
| H | -3.06322 | -1.5023 | -1.08678 |
| H | -3.21505 | -2.57563 | 0.278765 |
| H | -5.06862 | -1.09455 | 0.395688 |
| H | -3.98063 | -0.57902 | 1.682442 |
| H | -3.16391 | 2.028935 | 1.528259 |
| H | -4.12274 | 0.523651 | -2.12965 |
| H | -5.70788 | 0.846483 | -1.39647 |
| H | 4.908167 | 0.667247 | -0.53417 |
| C | 5.498213 | 2.858061 | 0.580999 |
| O | 5.888216 | 1.917775 | -0.09088 |
| O | 4.288335 | 3.020289 | 1.042988 |
| H | 6.163531 | 3.682023 | 0.865162 |
| H | 3.707896 | 2.248486 | 0.763656 |

Conf13 (-1344.328784 Hartree)

| Symbol | X | Y | Z |
| --- | --- | --- | --- |
| C | 0.747754 | -1.84706 | -2.73288 |
| C | -0.53106 | -1.57905 | -1.94111 |
| C | -0.35993 | -1.76476 | -0.42057 |
| C | 0.862753 | -0.9032 | 0.034934 |
| C | -1.60001 | -1.14431 | 0.334034 |
| C | -1.37043 | -1.24415 | 1.826529 |
| C | -0.1721 | -0.46351 | 2.302066 |
| C | 1.071454 | -0.94783 | 1.550746 |
| C | 2.19228 | -1.10117 | -0.76277 |
| C | 1.891006 | -0.94863 | -2.27571 |
| C | -0.26065 | -3.26142 | -0.09147 |
| C | 2.929739 | -2.41385 | -0.48396 |
| C | 3.126706 | 0.048409 | -0.38216 |
| O | -0.43534 | 0.91994 | 2.049901 |
| C | -2.06185 | -2.0004 | 2.678513 |
| C | -2.96923 | -1.6501 | -0.1292 |
| C | -4.16075 | -0.81293 | 0.384387 |
| C | -4.08992 | 0.629029 | -0.00418 |
| C | -4.31852 | 1.204632 | -1.19122 |
| C | -4.10815 | 2.653099 | -1.07339 |
| O | -4.20891 | 3.524813 | -1.89852 |
| O | -3.7502 | 2.923707 | 0.219197 |
| C | -3.7152 | 1.707831 | 0.964103 |
| O | 4.270248 | -0.12794 | 0.026269 |
| O | 2.607875 | 1.247784 | -0.54354 |
| H | 0.55806 | -1.66722 | -3.79632 |
| H | 1.032948 | -2.90199 | -2.65477 |
| H | -1.32395 | -2.23679 | -2.31061 |
| H | -0.85748 | -0.54852 | -2.13669 |
| H | 0.564923 | 0.125724 | -0.19701 |
| H | -1.55697 | -0.07539 | 0.08729 |
| H | -0.03419 | -0.62213 | 3.379327 |
| H | 1.307641 | -1.96011 | 1.89387 |
| H | 1.925107 | -0.32254 | 1.844076 |
| H | 2.800514 | -1.16037 | -2.84891 |
| H | 1.621095 | 0.093206 | -2.47539 |
| H | 0.018769 | -3.44611 | 0.947425 |
| H | -1.22874 | -3.74389 | -0.25514 |
| H | 0.456523 | -3.77968 | -0.72779 |
| H | 2.374391 | -3.27063 | -0.86034 |
| H | 3.904409 | -2.40286 | -0.97567 |
| H | 3.106295 | -2.56448 | 0.582711 |
| H | 0.377817 | 1.412857 | 2.196991 |
| H | -2.8954 | -2.62184 | 2.369704 |
| H | -1.80141 | -2.02897 | 3.732517 |
| H | -3.00535 | -1.63694 | -1.22096 |
| H | -3.13298 | -2.68935 | 0.172613 |
| H | -5.07953 | -1.2435 | -0.02598 |
| H | -4.23054 | -0.88785 | 1.472326 |
| H | -4.61638 | 0.737086 | -2.11909 |
| H | -4.43167 | 1.776506 | 1.790511 |
| H | -2.71261 | 1.568429 | 1.383932 |
| H | 3.27097 | 1.952145 | -0.29096 |
| C | 5.47207 | 2.91581 | 0.516257 |
| O | 4.336792 | 3.144476 | 0.133479 |
| O | 6.007332 | 1.735083 | 0.66669 |
| H | 6.167631 | 3.722138 | 0.77829 |
| H | 5.341862 | 1.021575 | 0.418441 |
